# Selective Functional Network Changes Following tDCS-Augmented Language Treatment in Primary Progressive Aphasia

**DOI:** 10.3389/fnagi.2021.681043

**Published:** 2021-07-12

**Authors:** Yuan Tao, Bronte Ficek, Zeyi Wang, Brenda Rapp, Kyrana Tsapkini

**Affiliations:** ^1^Department of Cognitive Science, Johns Hopkins University, Baltimore, MD, United States; ^2^Department of Neurology, Johns Hopkins School of Medicine, Baltimore, MD, United States; ^3^Division of Biostatistics, School of Public Health, University of California, Berkeley, Berkeley, CA, United States; ^4^Department of Psychological and Brain Sciences, Johns Hopkins University, Baltimore, MD, United States; ^5^Department of Neuroscience, Johns Hopkins School of Medicine, Baltimore, MD, United States

**Keywords:** primary progressive aphasia, fronto-temporal dementia, resting state – fMRI, network analysis, transcranial direct current stimulation

## Abstract

**Objective:**

Transcranial direct current stimulation (tDCS) has shown promising results when used as an adjunct to behavioral training in neurodegenerative diseases. However, the underlying neural mechanisms are not understood and neuroimaging evidence from pre/post treatment has been sparse. In this study, we examined tDCS-induced neural changes in a language intervention study for primary progressive aphasia (PPA), a neurodegenerative syndrome with language impairment as the primary clinical presentation. Anodal tDCS was applied to the left inferior frontal gyrus (LIFG). To evaluate the hypothesis that tDCS promotes system segregation, analysis focused on understanding tDCS-induced changes in the brain-wide functional network connectivity of the targeted LIFG.

**Methods:**

Resting-state fMRI data were obtained from 32 participants with PPA before and after receiving a written naming therapy, accompanied either by tDCS or sham stimulation. We focused on evaluating changes in the global connectivity of the stimulated LIFG-triangularis (LIFG-tri) region given its important role in lexical processing. Global connectivity was indexed by the graph-theoretic measure *participation coefficient* (PC) which quantifies a region’s level of system segregation. The values before and after treatment were compared for each condition (tDCS or Sham) as well as with age-matched healthy controls (*n* = 19).

**Results:**

Higher global connectivity of the LIFG-tri before treatment was associated with greater dementia severity. After treatment, the tDCS group showed a significant decrease in global connectivity whereas the Sham group’s did not change, suggesting specific neural effects induced by tDCS. Further examination revealed that the decrease was driven by reduced connectivity between the LIFG-tri and regions outside the perisylvian language area, consistent with the hypothesis that tDCS enhances the segregation of the language system and improves processing efficiency. Additionally, we found that these effects were specific to the LIFG-tri and not observed in other control regions.

**Conclusion:**

TDCS-augmented language therapy in PPA increased the functional segregation of the language system, a normalization of the hyper-connectivity observed before treatment. These findings add to our understanding of the nature of tDCS-induced neural changes in disease treatment and have applications for validating treatment efficacy and designing future tDCS and other non-invasive brain stimulation (NIBS) treatments.

## Introduction

Primary progressive aphasia (PPA) is a neurodegenerative syndrome with relatively early onset (ages 50–60) in which language impairment is the primary clinical presentation (Grossman, 2010; Mesulam et al., 2014). Thus far, there is no specific treatment for this devastating disease, and the underlying neuropathologies and genetic bases are still under active investigation. Recently, non-invasive brain stimulation (NIBS) has shown promising results in mitigating language symptoms in PPA [see review by Pini et al. (2018) and Tippett et al. (2015)], and in particular, transcranial direct current stimulation (tDCS) combined with behavioral therapy has been shown to produce reliable benefits at low cost with a high safety profile (Cotelli et al., 2014, 2016; Tsapkini et al., 2014, 2018; Hung et al., 2017; Roncero et al., 2017; Ficek et al., 2018).

While the most critical outcome measure of tDCS effectiveness is behavioral change, markers of the brain’s physiological responses are also important for assessing its effects. Such markers may be especially relevant because behavioral changes can be subtle and difficult to evaluate owing to factors such as dosage and disease progression. One commonly used neural measure has been resting-state functional connectivity (RSFC), often obtained with functional MRI, that requires no task to be administered during fMRI scanning. Another important feature of RSFC is that it can be used to evaluate the large-scale functional network organization of the brain, which has been increasingly recognized as being relevant to understanding neurodegenerative diseases (Seeley et al., 2009; Fornito et al., 2015). In this regard, it is noteworthy that neural stimulation techniques, including tDCS, have been shown to induce network-wide effects [see review by Sale et al. (2015)]. However, there is little understanding of the mechanisms underlying the network-level changes associated with tDCS, in part due to the scarcity of longitudinal network analyses in clinical populations [see review by Pini et al. (2018)].

In PPA, tDCS-induced functional connectivity (FC) changes have, thus far, only been reported by our group (Ficek et al., 2018). In a study reporting significant tDCS-induced behavioral improvements in language performance, Ficek et al. (2018) examined the RSFC FC strength between the (anodal) stimulation site – the left inferior frontal gyrus (LIFG) – and several regions of interest (ROIs) in the frontal, parietal, and temporal lobes, and found that tDCS (relative to Sham stimulation) reduced the FC strength between the stimulated LIFG and other temporal and parietal lobe ROIs. The finding of tDCS-related reduction in FC points to the possibility that tDCS induces “functional decoupling” between different neural sub-systems, which serves to enhance system segregation and increase the independence of each system. To specifically investigate the hypothesis that anodal tDCS enhances system segregation of the LIFG, in the current study we build on these previous findings in Ficek et al. (2018) to carry out a more comprehensive investigation of whole-brain network changes associated with tDCS, focusing on functional segregation and integration both within and between networks.

### System Segregation and Modularity in Healthy and Diseased Brains

The concept of modularity (i.e., system segregation) has a long history in the development of our understanding of how the brain processes information. More recently, with advances in neuroimaging techniques, this notion has been supported by the finding that the brain has a modular functional organization consisting of distributed, minimally overlapping large-scale networks (e.g., Bullmore and Sporns, 2009; Power et al., 2011; Thomas Yeo et al., 2011. Note that those large-scale networks are also referred to as “resting-state networks,” “modules,” “communities,” etc. Here we will refer to them as “modules.” Furthermore, although the brain’s anatomical organization has also been found to be modular, here we focus on the brain’s functional organization). Although the specification of the individual functional modules is still an active research topic, there is general agreement that primary sensory-motor processing areas (e.g., sensorimotor cortex and primary visual cortex) form modules that carry out specialized, domain-specific functions (Biswal et al., 1995; Bertolero et al., 2015; Thomas Yeo et al., 2015), and that so-called “association cortex” is composed of multiple modules (e.g., *default-mode network*, *frontoparietal network*, etc.) that are involved in high-level functions such as executive control, task switching, and so on (Dosenbach et al., 2007; Cole et al., 2013).

One key advantage of a modular organization is its efficiency in processing complex information by maintaining an optimal balance between flexibility and wiring costs (Bullmore and Sporns, 2012; Sporns, 2013). Specifically, a modular organization consists of segregated modules (i.e., groups of tightly inter-connected nodes) with sparse long-distance connection between modules. Within such a topographical scheme, segregated sub-systems/modules can perform tasks with maximal proficiency and automaticity while minimizing wiring costs. Costly global interactions, which are critical to global coordination and flexibility, are held to a minimum. Empirical evidence supporting these organizational principles has been obtained from functional neuroimaging studies with healthy participants. For instance, higher functional segregation, or modularity, has been linked to greater processing proficiency, e.g., when the task demand is lower (Kitzbichler et al., 2011; Braun et al., 2015) and after a novel task had been learned (Bassett et al., 2011, 2015; Mohr et al., 2016). Additionally, reduced modularity and associated behavioral deficits have been reported in neurological conditions, including stroke (Siegel et al., 2018), Alzheimer’s disease (Brier et al., 2012, 2014) as well as age-related decline (Chan et al., 2014; Geerligs et al., 2015). In sum, a modular neural organization supports efficient information processing, and pathological changes to the organization can have significant behavioral consequences.

Although the concept of modularity is typically applied to the global topology of the brain, it emerges from, and can be evaluated at, the regional level. For instance, reduced global modularity can be caused by decreased within-module connectivity, increased between-module connectivity, or both. Moreover, different diseases may selectively target specific networks (e.g., *default-mode network* in Alzheimer’s disease; Seeley et al., 2009). Therefore, regional characteristics may be particularly important for understanding network changes in disease and for developing effective NIBS treatments.

### Current Study

The goal of the current study was to understand tDCS-induced changes affecting the functional segregation/integration of the stimulation site – the LIFG – in a cohort of individuals with PPA receiving concurrent language therapy. When combining tDCS and behavioral therapy, one important issue is the pairing between the task and the simulation location. Specifically, it has been argued that, in order for tDCS to maximally affect neuronal processes, the stimulated region should be actively recruited by the behavioral task that participants engage in during the stimulation (Stagg and Nitsche, 2011; Bikson and Rahman, 2013). Therefore, in the current study, we aimed to target the anterior/ventral part of the LIFG [i.e., triangularis, or BA45, referred to as LIFG-triangularis (*LIFG-tri*) hereinafter], as this region plays a critical role in lexical semantic knowledge retrieval (Bookheimer, 2002; Devlin et al., 2003; Binder et al., 2009), a key cognitive process targeted in the language therapy (written naming) used in this study. Previous studies that also applied anodal tDCS to the LIFG paired with a semantic task showed improved performance along with neural changes in the anterior/ventral LIFG in healthy participants as well as individuals with mild cognitive impairment (MCI) (Holland et al., 2011; Meinzer et al., 2012, 2013, 2015).

In terms of data analysis, the approach was as follows: First, in order to evaluate system segregation, we defined a reference modular organization on the basis of the healthy control (HC) data which corresponded to the division of the whole-brain functional connectome into a set of non-overlapping sub-systems/modules. Second, on the basis of those reference modules, we calculated the level of global connectivity diversity for the LIFG-tri using the network measure *participation coefficient* (or PC, Guimerà and Amaral, 2005). Specifically, PC quantifies the extent to which a node’s connections are evenly distributed across all the modules such that if all of a node’s connections are within one module, the node’s PC value equals zero, whereas if the connections are evenly distributed across all the modules, the node’s PC value approaches one. In other words, nodes with high PC values are highly integrated with other modules (thus are often called as the “connector hubs”), while nodes with low PC are rather isolated from other modules. Third, as PC only provides a single summary statistic, we also examined the underlying distributions of connections for the LIFG-tri, focusing on *within-module* and *between-module* connectivity, i.e., LIFG-tri connections within its own module and connections between the LIFG-tri and other modules. We hypothesized that the *within/between-module* property is relevant not only because it is closely related to the PC measure, but also because the balance of within and between-module connectivity is a key feature of global modular organization and system segregation. Fourth, in order to evaluate the degree to which the tDCS effects were regionally specific, we also examined the FC profiles of several control ROIs using the same network measures. We examined the right-hemisphere homolog of the LIFG-tri given the strong structural and functional connectivity between homologs. The other reason that the IFG triangularis (RIFG-tri) region was selected as a control region is the long-standing interest in understanding the role of the right-hemisphere homolog subsequent to left-hemisphere damage. Findings in this regard have been mixed with some researchers finding that the right-hemisphere plays a compensatory role to support language functions subsequent to left-hemisphere stroke, while others have found that it plays a maladaptive role (Saur et al., 2006; Turkeltaub, 2015). The second control ROI was the right precuneus. We expected this to be a “neutral” ROI, as this region is relatively spared in PPA and considered to belong to a different functional network than the LIFG-tri (the *default-mode network*). Thus, the right precuneus would allow us to evaluate if tDCS-induced FC changes occur brain wide or are more spatially specific. Finally, we also examined the other two LIFG subdivisions, the *orbitalis* and the *opercularis*, given that they were within the stimulation range.

In sum, in order to further our understanding of the neural changes associated with tDCS effects, this study examined the global FC profile of the stimulation target LIFG-tri before and after a tDCS-augmented naming therapy, with comparisons to a sham-stimulation group as well as to age-matched HCs. Moreover, we examined four “control” regions (RIFG-tri, right precuneus, and the orbitalis and opercularis subdivisions of the LIFG) to investigate whether tDCS-induced FC changes were specific to the targeted LIFG-tri.

## Materials and Methods

### Participants

**FIGURE 1:**
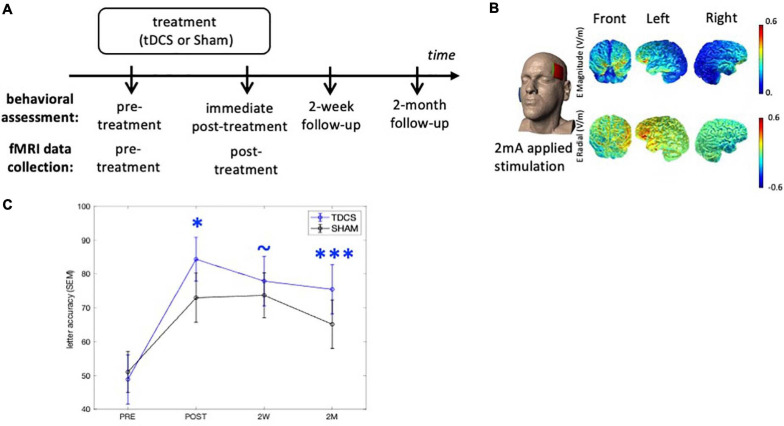
Experimental design and tDCS current flow modeling. **(A)** Behavioral outcomes were assessed at three time-points after treatment. The neuroimaging data were collected at the pre- and immediate post-treatment time-points. **(B)** The anodal electrode was placed on the left frontal lobe (F7) and the cathodal electrode was on the right cheek (current flow estimation image courtesy of Dr. Marom Bikson). **(C)** Behavioral outcomes. TDCS consistently shows greater treatment gain of the treated items over Sham after treatment. ^∗^*p* < 0.05, ^∗∗∗^*p* < 0.0001, ∼*p* < 0.1. SEM, standard error of the mean.

Resting-state fMRI data were collected from 32 participants with PPA (16 females, mean age = 67, SD = 6.73) before and after a tDCS-augmented language therapy as part of a clinical trial (NCT02606422). Half of the participants (*n* = 16) received anodal tDCS over the LIFG and half received sham (*n* = 16), and both treatments were coupled with behavioral language intervention (Figure 1A).^1^ This cohort included the three PPA variants (non-fluent, logopenic, and semantic) and, for each variant, similar numbers of participants received tDCS or sham (10 non-fluent variant: 4 received tDCS, 14 logopenic variant: 8 received tDCS, 8 semantic variant: 4 received tDCS). All participants had a history of progressive language deficits without other etiology (e.g., stroke, tumors, etc.) or primary memory deficits. Differential diagnosis was based on three types of evidence: neuropsychological and language testing, MRI, and clinical assessment, according to criteria in Gorno-Tempini et al. (2011) (see also Neophytou et al., 2019; Themistocleous et al., 2020). Demographic and clinical information for the participants is reported in Table 1. The participants were randomly assigned to the tDCS and the Sham groups, after which it was determined that the two groups did not differ with regard to overall clinical dementia rating for the revised Fronto-Temporal Lobar Degenerations Clinical Dementia Rating (FTLD-CDR, Knopman et al., 2008) and the language sub-component. The overall dementia scores were independently calculated by three raters based on information collected from the participant and family, language and cognitive testing, and questionnaires. The raters then convened to discuss and produce a consensus score. In addition, as in this study we were targeting the LIFG-tri, we also examined whether the tDCS and the Sham group differed in other language functions that have been closely related to the LIFG-tri (i.e., semantic fluency, naming, syntactic processing). As shown in Supplementary Figure 5, we found no difference between the two groups in those assessments.

**TABLE 1:** Demographic characteristics of the healthy controls (HC) and the two PPA treatment groups, the group mean and standard deviation values are shown. The two treatment groups were compared with independent *t*-test, results shown in the last column.

	HC (*N* = 19)	PPA tDCS (*N* = 16)	PPA Sham (*N* = 16)	tDCS vs. Sham (*t*-Value)
Age	65 (8.14)	64 (7.45)	69 (5.06)	−2.17*
Gender (*N* of female)	14	8	8	NA
Education (year)	16 (2.63)	17 (2.02)	17 (1.91)	0.23
Time since onset (year)	NA	5.13 (3.55)	4.22 (2.36)	0.85
FTDL-CDR (scale 0–15)	NA	7.31 (3.93)	7.84 (5.52)	−0.32
FTDL-CDR Language (scale 0–3)	NA	1.88 (0.87)	1.88 (0.81)	0

*p < 0.05.

MRI data were also collected from a group of age-matched HCs (*n* = 19, 15 females, mean age = 65, SD = 7.81, see Table 1). All control participants were right-handed and native English speakers. The study was approved by the Johns Hopkins Hospital and Johns Hopkins University Institutional Review Board. All participants provided informed consent.

### Experimental Design and Behavioral Intervention

The design and tDCS methods have been reported in our previous publication (Tsapkini et al., 2018) and hence here we summarize the key information relevant to the current study. The behavioral effects of this clinical trial have been described in Tsapkini et al. (2018) in which data from a total 36 PPA participants were analyzed. Eleven participants in this previous study were not included in the current one because the MRI data were not collected due to health issues (pacemakers, claustrophobia, and other medical conditions), and data from seven additional participants who were subsequently evaluated was included in the current participant group. For a comprehensive report of the behavioral results, see Tsapkini et al. (2018).

#### Experimental Design

Participants were recruited from Johns Hopkins clinics and referrals from diagnostic centers. All participants received anodal tDCS or sham over the LIFG paired with a written naming/spelling therapy (see section “Written Word Production Intervention”), with stimulation conditions assigned using a stratified randomization scheme within each variant. The participants received a maximum of 15 sessions of daily therapy (mean 11.6 ± 1.9). Behavioral measures were collected at multiple time-points and the brain imaging data were collected before and immediately after treatment (Figure 1A). Specifically, the interval between the imaging collection and intervention was between 0 and 3 days, both before and after treatment.

#### Written Word Production Intervention

The behavioral intervention in this study targeted written word production. For each participant, a set of treatment words were individually selected with accuracy ranging from 20 to 60% (10–30 trained and 10–30 untrained words per participant). The written letter accuracy outcome measure was calculated following the Goodman and Caramazza (1986) scoring system. The specific training approach was based on CART (Beeson and Egnor, 2006; Rapp and Glucroft, 2009) as follows: The participant was shown a picture on the computer, asked to orally name it, and then to write the name. If they could not name the picture (orally or in writing), they were asked to describe it to reinforce semantic knowledge, as in semantic feature analysis treatment (Boyle, 2001). If they still could not produce the word orally, they were provided with the correct word and asked to repeat it three times. Likewise, if the patient could not write the word or wrote it incorrectly, the clinician provided the correct word, reviewed each letter’s sound, then asked the patient to copy the word three times.

### tDCS Methods

The tDCS methods have been previously reported in detail (Ficek et al., 2018; Tsapkini et al., 2018) and are summarized here (Figure 1B). Anodal tDCS was delivered by a Soterix 1 × 1 CT device and was applied to the left frontal lobe corresponding to the F7 electrode using the EEG 10–20 electrode position system (Homan et al., 1987). The reference electrode, the cathode, was placed on the right cheek given that extracephalic placement of the reference electrode has been shown to improve current density and delivery (Russell et al., 2017). Current was delivered at 2 mA intensity (estimated current density 0.08 mA/cm^2^) for 20 min in the tDCS condition. Non-metallic, conductive rubber electrodes covered with saline-soaked 5 cm-by-5 cm sponges were used to minimize the possibility of chemical reactions at the skin/electrode interface. For both tDCS and sham interventions, the electrical current was ramped up at stimulation onset, eliciting a transient (typically 30 s) tingling sensation. In the sham condition, after ramping up, current intensity was decreased to 0 mA. Both active tDCS and sham conditions lasted for 20 min. Behavioral therapy started at the same time as the simulation (for both conditions) and continued for another 20–25 min. Both the therapist and participant were blind to the stimulation condition. Participants were asked to report their general pain level once or twice during each session with the Wong-Baker FACES Pain Rating Scale.^2^

### MRI Imaging Data Acquisition

All MRI data were collected using a Phillips 3T scanner at the F.M. Kirby Research Center for Functional Brain Imaging (Baltimore, MD, United States). The scanning protocol of each PPA participant included one session of resting-state and multiple structural scanning protocols, including a T1-weighted structural image included in this analysis (see Ficek et al., 2018 for further details). For all but 10 PPA participants, the resting-state fMRI (rs-fMRI) scan lasted 8.75 min (210 data-points), and for the remaining 10 participants the scan lasted 6.5 min (156 data-points). The acquisition parameters of the rs-fMRI were as follows: TR = 2500 ms, TE = 30 ms, FOV = 240 mm × 141 mm × 240 mm (ap, fh, rl), flip angle = 75°, voxel dimension = 3 mm × 3 mm × 3 mm, data matrix = 80 × 80 × 47. The T1-weighted structural MRI acquisition parameters were as follows: TR = 8.1 ms, TE = 3.7 ms, FOV = 224 mm × 160 mm × 180 mm (ap, fh, rl), flip angle = 8°, voxel dimension = 1 mm × 1 mm × 1 mm, data matrix = 224 × 224 × 160.

For the HCs, seven underwent the same scanning protocol as described just above for the PPA participants. The other 12 were scanned with a slightly different protocol as they were recruited for a different experiment: Two 7-min runs of rs-fMRI (175 time-points) were carried out consecutively, the acquisition parameters were as follows: TR = 2400 ms, TE = 20 ms, FOV = 206 mm × 123 mm × 220 mm (ap, fh, rl), flip angle = 90°, voxel dimension = 1.7 mm × 1.7 mm × 3 mm, data matrix = 128 × 128 × 41. The T1-weighted structural MRI acquisition parameters were: TR = 6 ms, TE = 2.9 ms, FOV = 256 mm × 256 mm × 176 mm (ap, fh, rl), flip angle = 9°, voxel dimension = 1 mm × 1 mm × 1 mm, data matrix = 256 × 256 × 176. Note that despite some minor differences, both protocols used with the HCs included standard parameters for structural MRI and rs-fMRI acquisition.

### MRI Imaging Data Processing and Functional Connectivity Calculation

As described in Ficek et al. (2018) and Tao et al. (2020), the MRI data preprocessing and FC calculations were carried out with MRICloud,^3^ a publicly accessible cloud-based platform for automatic neuroimaging data analysis (Mori et al., 2016). MRICloud provides standardized data processing service with pre-tuned parameters, therefore here we only summarize the preprocessing procedure as provided by the developer. For details of the implementation, we refer the readers to the published work by the development team (Faria et al., 2012; Mori et al., 2016). First, the T1 structural image of each individual (in native space) was parcellated into 283 anatomical structures (atlas version “Adult50_90yrs_283Labels_19atlases_M2_V9B”^4^), from which 76 gray matter structures were selected to construct the functional connectome (see below). The parcellation was conducted with a multi-atlas fusion label algorithm (MALF) and large deformation diffeomorphic metric mapping (LDDMM), an algorithm that minimizes mapping error due to atrophy or local shape deformation (Faria et al., 2012).

The rs-fMRI images were preprocessed with MRICloud’s rs-fMRI processing pipeline,^5^ which includes routines imported from SPM5. The preprocessing steps were as follows: slice-timing correction, motion correction that realigned the images to the first volume, physiological nuisance removal with CompCor (Behzadi et al., 2007), outlier volume rejection with “ART”.^6^ The functional images were then co-registered to each individual’s T1 scan with rigid-body transformation, and the averaged time-courses corresponding to 76 gray matter structures (38 in each hemisphere) were extracted, and pairwise correlation values (with Fisher’s z-transformed) were calculated to create a 76 × 76 symmetrical connectivity matrix for each participant (and each time-point). We refer to the 76 gray matter structures as “nodes” in the following network analyses.

### Identifying the Reference Modular Organization

In the current study we focused on the network measure, PC (Guimerà and Amaral, 2005), which quantifies a node’s connectivity diversity across multiple modules. Thus, to calculate PC, first we identified an aprior reference modular organization based on the HC group. Specifically, we carried out hierarchical clustering (Ward’s criterion, MATLAB implementation) using the averaged functional correlation matrix of the HC group. Note that the clusters (i.e., modules) identified in this way are often called “resting-state functional networks (RSNs)” or “communities” in the literature (e.g., Smith et al., 2009; Power et al., 2011). Although they can be calculated with different methods (e.g., clustering, ICA) these terms all refer to groups of brain regions with highly correlated time-series. In this manuscript we will refer to these functionally defined clusters of brain regions as *modules*.

### Measuring Global Connectivity

#### Network Measure Calculation

The network measure PC is a measure calculated for each node that quantifies the connectivity diversity of that node (Guimerà and Amaral, 2005, Eq. 1). All network analyses were carried out with the MATLAB toolbox *Brain Connectivity Toolbox* (Rubinov and Sporns, 2010).

(1) $$PC_{i}=1-\sum_{m\in M}{\left(\frac{k_{i}\left(m\right)}{k_{i}}\right)}^{2}$$

Participation coefficient of node *i*. *k*_*i*_ is the number of total connections of node *i* (i.e., degrees), *k*_*i*_(*m*) is the number of connections with module *m*, *M* is the set of all modules.

**FIGURE 2:**
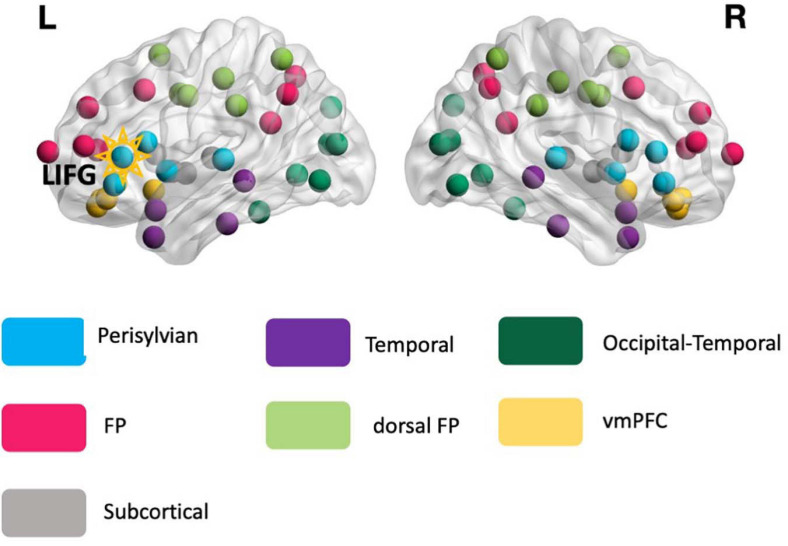
The reference modular organization. The reference modular organization was identified from the rs-fMRI data of the healthy controls and served as the basis for calculating the global connectivity measure *participation coefficient* (PC, see Eq. 1) for all participants. Each node in the image corresponds to 1 of the 76 anatomical structures. The LIFG-triangularis – the stimulation target (highlighted) – is situated in the perisylvian module (turquoise). FP, frontoparietal; dFP, dorsal frontoparietal; vmPFC, ventromedial prefrontal cortex.

To calculate PC values for each node, first, the connectivity matrix of each individual participant was converted to an undirected binary graph by preserving the strongest (thresholded) connections across the graph and then binarizing their connectivity strengths to 1’s or 0’s. As there is not an agreed-upon cut-off threshold value for the “strongest connections,” we applied a range of proportional threshold values (top 5, 10, 20, 25, 30, 40% of the connections) to obtain binary graphs. Then, on the basis of the reference modular organization described in Section “Identifying the Reference Modular Organization” (see section “The Reference Modular Organization” in Result, Figure 2), the PC value of each node was calculated at each threshold. Finally, we averaged the PC values across the proportional thresholds for use in all subsequent analyses.

#### Statistical Analyses on the Network Measure *Participation Coefficient*

Here we describe the statistical analyses applied to the global connectivity measure PC of the main ROI LIFG-tri, and the four control ROIs: RIFG-tri, right precuneus, LIFG-opercularis, LIFG-orbitalis. Note that as the PPA participants were randomly assigned to the treatment groups (tDCS and Sham), for the evaluation of the pre-treatment data we combined the data of both treatment groups (*N* = 32). The statistical analyses consisted of two steps. First, we compared the ROI’s pre-treatment PC values of the PPA group to the values of the HCs, and also calculated the relationship between the PC values and the overall dementia severity (FTDL-CDR, Knopman et al., 2008). This pre-treatment analysis provided the basis for later characterizing treatment-related neural changes relative to normal levels (i.e., whether treatment-based changes involved movement toward or away from the normal pattern). The group comparison between PPA and the HC group and the relationship between PC and the dementia scores were carried out with general linear regression modeling in R (*lm* function) including the co-variates: age, years of education, gender, and in-scanner motion (measured as the *root-mean-square* of the six motion parameters).

Second, we examined whether the tDCS group exhibited distinct pre- to post-treatment changes from the Sham group. To do so, we evaluated a fixed-effects model with R (*lm* function), with the PC changes (post minus pre) of each participant as the dependent variable, treatment group (tDCS vs. Sham) as the key predictor variables, and the following co-variates: overall dementia severity (FTDL-CDR), age, years of education, gender, in-scanner motion. Given significant differences between tDCS and Sham, the pre- and post-treatment PC values in each group were compared with paired *t*-tests to examine how the PC values changed in each treatment group.

Finally, we also examined the relationship between the PC changes and treatment-related behavioral changes. Similar to the above analyses, we calculated a fixed-effects model with the dependent variable corresponding to the improvement score at the 2 month follow-up (given that the augmentative effect of tDCS over Sham was the largest at this time-point) (Figure 1C). The independent variables consisted of the PC changes, treatment group, overall dementia severity (FTDL-CDR), age, years of education, gender, and the interaction term between PC changes and treatment-group to evaluate whether the two groups showed different relationships between neural and behavioral changes.

#### Examining *Within-Module* and *Between-Module* Connectivity of the LIFG (Triangularis)

Given that the graph-theoretic measure PC only quantifies a node’s connectivity diversity across modules with a single value (Eq. 1), it is also of interest to examine the specific connectivity patterns that give rise to any overall pre-post treatment PC changes. To do so, we examined the *within-* and *between-module* connectivity for the target ROI LIFG-tri: The former refers to the connections between the LIFG-tri and the nodes that were in the same module, and the latter refers to the connections between the LIFG-tri and nodes in other modules (see Figure 2 and section “The Reference Modular Organization,” for the modular organization used in the analyses).

To quantify the *within-* and *between-module* connectivity, we simply counted the number of connections of the LIFG-tri within or outside its own module. Specifically, to be consistent with the PC calculation (section “Network Measure Calculation”), we counted the connections at each proportional threshold value that was used to construct the binary graphs, and then used the averaged values across the thresholds in subsequent analyses. Pre- to post-treatment connectivity changes were compared with paired *t*-tests and values of each time-point were compared to the HC group with independent *t*-tests. Furthermore, to evaluate whether any effects were restricted to a specific module/s, we evaluated separately the *between-module* connectivity of the LIFG-tri with every other module. The same statistical comparisons described above were carried out for each set of between-module connections and the results were corrected using the FDR procedure (Yekutieli and Benjamini, 1999).

Finally, we examined *within-* and *between-module* connectivity for each hemisphere separately following the same procedures, i.e., counting the number of connections between the LIFG-tri and other nodes in the left or right hemisphere, respectively (see section “The Reference Modular Organization” and Figure 2).

## Results

### Behavioral Results

The randomization procedures successfully blinded participants to the assigned stimulation condition since they were at chance (53% correct) at reporting whether they got active tDCS or sham at each period of stimulations in the original crossover study.

We examined the treatment effects in the current cohort following our previous study that reported on the treatment efficacy (Tsapkini et al., 2018). The augmentative effects of tDCS over Sham on the improvement scores for the trained words were evaluated at immediate post-treatment, 2-week, and 2-month follow-ups (Figure 1A), taking into account the co-variates of: pre-treatment accuracy, PPA variant, number of treatment sessions, sex, age, years post onset of symptoms, and clinical dementia rating (FTDL-CDR) and its language sub-score. At the immediate post-treatment time-point, tDCS showed a significant augmentative effect over Sham (*p* = 0.03), which was maintained at 2-month follow-up (*p* = 0.0002). The behavioral effects of each group are depicted in Figure 1C and details of the analysis and statistics are reported in Supplementary Material 1. Although a few of the participants reported in Tsapkini et al. (2018) were not included in the current analysis due to the lack of a full imaging dataset, the behavioral results for the participants in the current study were consistent with the previous Tsapkini et al. (2018) report.

### The Reference Modular Organization

As described earlier, the global connectivity measure PC is computed based on a reference modular network identified from the HCs. The reference modular organization consisted of seven modules (Figure 2): (1) temporal, (2) frontoparietal (FP), (3) dorsal frontoparietal (dFP), (4) ventromedial prefrontal (vmPFC), (5) occipital-temporal cortex, (6) subcortical, and (7) perisylvian. All the modules were bilateral and the LIFG-tri ROI, which served as the tDCS target, was situated within the perisylvian module.

### tDCS Effects on Global Connectivity of the LIFG-Triangularis

#### Global Connectivity of the LIFG-tri in PPA Before Treatment

**FIGURE 3:**
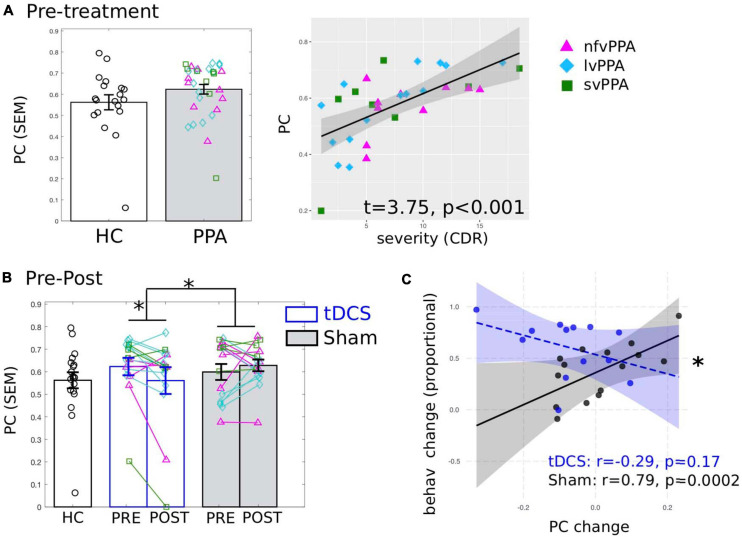
Global connectivity (*participation coefficient*, PC) of the tDCS stimulation target left inferior frontal gyrus, triangularis (LIFG-tri). **(A)** Pre-treatment PC values of the PPA participants (both tDCS and Sham group combined) compared to the healthy controls (HC). Left: PPA shows numerically higher PC than HC though the difference is not statistically significant. Right: Higher PC is associated with behaviorally measured dementia severity (FTDL-CDR: Higher values indicate greater severity). The plot depicts the partial residuals of the regression model (visualized with jtools in R). The three variants are visualized with different colors and markers. **(B)** Pre- to post-treatment changes of the tDCS group (blue) and the Sham group (white), values of the HC (black) are the same as in panel **(A)**. Left: The tDCS and the Sham group differ significantly in terms of pre- to post-treatment PC changes, driven by a significant PC decrease for the tDCS group with no change for the Sham group. The three variants are visualized with different colors and markers as in panel **(A)**. **(C)** Relationship between PC and behavioral changes. The plot depicts the partial residuals of the regression model (visualized with the package interactions in R). The tDCS and the Sham groups are indicated by blue and black circles, respectively. The *x*-axis shows the PC changes (Post minus Pre), with positive values indicating increase from pre- to post-treatment and vice versa. The *y*-axis shows the treatment-related behavioral changes measured as proportional of maximal gain, such that the maximum improvement value is 100%. ^∗^*p* < 0.05. SEM, standard error of the mean.

First, we examined whether the global connectivity (i.e., PC) of the LIFG-tri was affected by disease. Here we report the average PC values calculated across multiple proportional threshold values (see section “Network Measure Calculation”), noting that similar effects were also observed at each threshold value (Supplementary Figure 4). At the pre-treatment timepoint (Figure 3A), when compared to the HC group, the PPA group (tDCS and Sham Groups combined) showed numerically higher PC though the effects were not significant (*t* = 0.84, *p* = 0.4). Importantly, nonetheless, higher PC values for the PPA group were associated with higher dementia severity (FTDL-CDR, *t* = 3.75, *p* = 0.0009, Figure 3A), indicating that the higher global connectivity of the LIFG-tri might be a result of the disease. In addition, males had higher PC values than females (*t* = 2.29, *p* = 0.03), and no other variables showed significant effects (The full regression results were reported in Supplementary Materials 1).

#### Pre- to Post-treatment Changes of the LIFG-tri’s Global Connectivity

To identify tDCS-induced changes, first we compared the pre- to post-treatment changes in PC between the tDCS and the Sham group and found a significant difference in the magnitude of the changes between the two treatment groups (*t* = −2.38, *p* = 0.03, Figure 3B). None of the other variables (i.e., age, years of education, gender, dementia severity, in-scanner motion) showed significant effects (see Supplementary Materials 1). Furthermore, the difference was specifically driven by a significant PC decrease in the tDCS group [*t*(15) = −2.48, *p* = 0.0255]. In contrast, the Sham group’s PC values did not change from pre- to post-treatment [*t*(15) = 0.46, *p* = 0.651]. Overall, these results provide evidence that tDCS resulted in distinct neural changes compared to behavioral treatment alone, such that the connections of the LIFG-tri became less widely distributed across multiple modules after treatment. Furthermore, given the positive correlation between PC and dementia severity before treatment (reported above in section “Global Connectivity of the LIFG-tri in PPA Before Treatment”), the treatment-related decrease in PC values indicate a tDCS-induced normalization of these values for the LIFG-tri.

#### Relationship Between Connectivity Changes and Behavioral Improvement

We correlated the global connectivity (PC) changes (shown in Figure 3B) and the treatment-related behavioral changes at the 2-month follow-up, as we found that one notable benefit of tDCS over sham was in maintaining the treatment gains (Figure 1C, also see Tsapkini et al., 2018). As shown in Figure 3C, there was a significant interaction (*t* = −2.48, *p* = 0.02) between the two groups such that for the tDCS group, greater PC decrease (greater normalization) was associated with greater treatment gain (*r* = −0.29, *p* = 0.17), whereas the Sham group showed the opposite pattern (*r* = 0.79, *p* = 0.0002). Regarding other variables, the FTDL-CDR scores were negatively correlated with behavioral changes such that greater overall severity was correlated with less improvement (*t* = −2.75, *p* = 0.01, see Supplementary Table 3). This result indicates that the PC decrease observed in the tDCS group was indeed behaviorally beneficial. The difference in the direction of the correlation between the two groups also suggests that the neural mechanisms supporting behavioral changes in the tDCS condition may be distinct from those associated with behavioral treatment alone (the Sham group).

In addition, as the tDCS and the Sham groups showed a significant difference in age (Table 1), to make sure the effects regarding the LIFG-tri reported here were not simply due to this age difference, we repeated the analyses with a subset of the participants who were matched in age (13 tDCS and 14 Sham). We found the effects reported above (section “Global Connectivity of the LIFG-tri in PPA Before Treatment”, “Pre- to Post-treatment Changes of the LIFG-tri’s Global Connectivity,” and “Relationship Between Connectivity Changes and Behavioral Improvement”) remained the same (Supplementary Material 4).

#### The Neurotopographic Distribution of the LIFG-tri’s Connections

**FIGURE 4:**
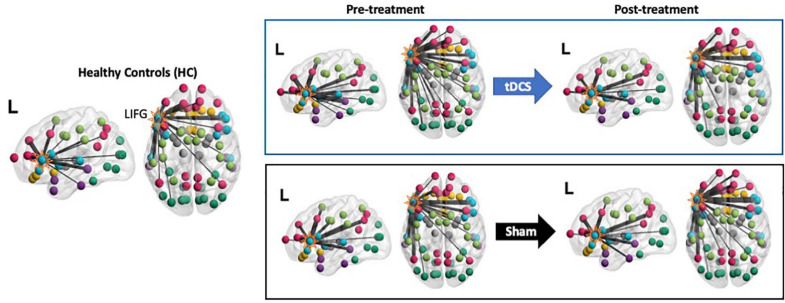
Visualization of the LIFG-triangularis’ connections for each group and time-point. The LIFG-tri is highlighted. The nodes are color-coded by their module membership as shown in Figure 2. Connection thickness indicates consistency across the proportional thresholds, such that thicker lines indicate stronger connections (see section “Network Measure Calculation” for the procedure for constructing binary graphs). A more diverse distribution of the connections across the modules corresponds to higher participation coefficient(PC) and, conversely, if the connections are more concentrated within certain module(s), PC values will be lower (see Eq. 1).

**FIGURE 5:**
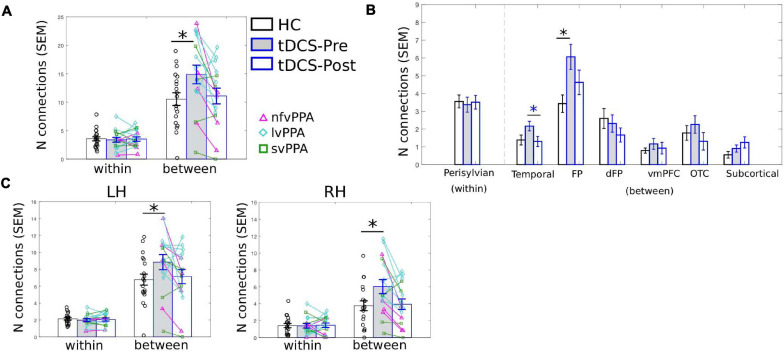
LIFG-triangularis (LIFG-tri) changes in *within-module* and *between-module* connectivity of the tDCS group at pre- and post-treatment time points. The healthy control group (HC) is indicated by black, and the tDCS group before and after treatment by blue frames with gray and white fill, respectively. The three PPA variants are visualized with different colors and markers. ^∗^*p* < 0.05. SEM, standard error of the mean. **(A)** The number of within- and between-module connections of the LIFG-tri for the HC and the PPA groups before and after tDCS treatment. There were no time-point or group differences in terms of *within-module* connectivity. With regard to *between-module* connectivity, at pre-treatment, the tDCS group had more connections than the HC group, a difference that disappeared after treatment. **(B)** LIFG-tri connections by module. The within-module (perisylvian module) values are the same as in panel **(A)**. For the between-module connections, a similar pattern of pre- to post-treatment normalization was observed for multiple modules, especially for the temporal and FP modules. **(C)** Numbers of within- and between-module connections of the LIFG-tri in each hemisphere. Individual hemisphere results were similar to the whole-brain results shown in panel **(A)**.

As the PC measure is a single statistic that summarizes a node’s global connectivity (Eq. 1), to understand the impact of the tDCS intervention on individual connections, we visualized the connection distributions underpinning the reported PC values. As shown in Figure 4, compared to the HCs, at pre-treatment, the PPA group (both the tDCS and the Sham subgroups) had more and stronger connections across multiple modules. This pattern corresponds to the high PC values in the PPA group (see Figure 3A). After treatment, the tDCS group showed visibly reduced connectivity across multiple modules, whereas the Sham group did not show clear changes. To quantitatively assess the neurotopographically distributed changes of the LIFG-tri’s connections observed for the tDCS group, we calculated the number of the LIFG-tri connections within its own module *perisylvian* (i.e., *within-module* connectivity) and also the number of connections with other modules (i.e., *between-module* connectivity) before and after tDCS. We then compared those values to those of the HC group. As shown in Figure 5A, at pre-treatment, there were more *between-module* connections for the LIFG-tri in the tDCS than the HC group [*t*(33) = −2.24, *p* = 0.0317], whereas the two groups did not differ in terms of *within-module* connections [*t*(33) = 0.32, *p* = 0.75]. At post-treatment, the number of *between-module* connections in the tDCS group decreased to a similar level as in the HC Group and the *within-module* connections continued to be no different than the HC’s [*between*: *t*(33) = −0.31, *p* = 0.76; *within*: *t*(33) = 0.08, *p* = 0.94]. Pre-post treatment comparisons for the tDCS group also indicated decreased *between-module* connectivity although the difference did not reach significance [*within*: *t*(15) = 0.27, *p* = 0.79; *between*: *t*(15) = −1.6, *p* = 0.13]. Overall, the results indicate that the hyper-connectivity (PC) of the LIFG-tri observed at pre-treatment was driven by a larger number of connections to other modules outside the *perisylvian* module, and that the treatment-related decreases in PC were largely driven by a normalization of this *between-module* connectivity.

At a more fine-grained level, when we examined the *between-module* connections of the LIFG-tri with each individual module, we observed the pattern of hyper-connectivity at pre-treatment and decrease/normalization after tDCS, in multiple modules (Figure 5B). Specifically, the *frontoparietal* module (FP) showed the largest effect, such that the number of connections between the LIFG-tri and the FP module was significantly higher at pre-treatment (corrected *p* = 0.02) and decreased toward normal levels after treatment, although the effect was not significant after multiple comparisons correction (corrected *p* = 0.13). Similarly, the LIFG-tri also showed a trend toward hyper-connectivity with the *temporal* module at pre-treatment (corrected *p* = 0.16) and a significant pre to post-treatment normalization (corrected *p* = 0.03). These comparisons were FDR corrected and the statistics are reported in Supplementary Material 2.

Finally, we examined the LIFG-tri’s connections with nodes in each hemisphere (e.g., the *within-module* connections corresponded to the connections between the LIFG-tri and the other *perisylvian* nodes within the left or right hemisphere separately). We found that the pattern reported above with regard to bilateral LIFG-tri connectivity was similar for both hemispheres, that is, there was a larger number of *between-module* connections for the tDCS group than the HC group at pre-treatment, and the number decreased at post-treatment (Figure 5C). Also, as with the whole-brain results, no differences were found for the *within-module* connections [Figure 5C; Pre-treatment: LH: *within*: *t*(33) = 0.70, *p* = 0.49; *between*: *t*(33) = −1.90, *p* = 0.0657. RH: *within*: *t*(33) = 0.04, *p* = 0.96; *between*: *t*(33) = −2.28, *p* = 0.0292. Post-treatment: LH: *within*: *t*(33) = 0.37, *p* = 0.71; *between*: *t*(33) = −0.35, *p* = 0.73. RH: *within*: *t*(33) = −0.13, *p* = 0.90; *between*: *t*(33) = −0.22, *p* = 0.83]. In addition, the by-module results were also similar in each hemisphere (Supplementary Material 2 and Supplementary Figure 1).

In sum, we examined the connection distributions underlying the LIFG-tri’s global connectivity (PC) decrease following behavioral treatment augmented with tDCS. We found that the decrease was largely driven by a reduction of the LIFG-tri’s *between-module* connections (i.e., connections to modules outside LIFG-tri’s own module *perisylvian*, similarly in both the left and right hemispheres). In particular, the largest reductions were seen between the LIFG-tri and the *frontoparietal* (FP) and the *temporal* modules. Moreover, these changes corresponded to a normalization of the pre-treatment hyper-connectivity of the LIFG-tri’s *between-module* connectivity.

### tDCS Effects for Control ROIs

#### The Right IFG

**FIGURE 6:**
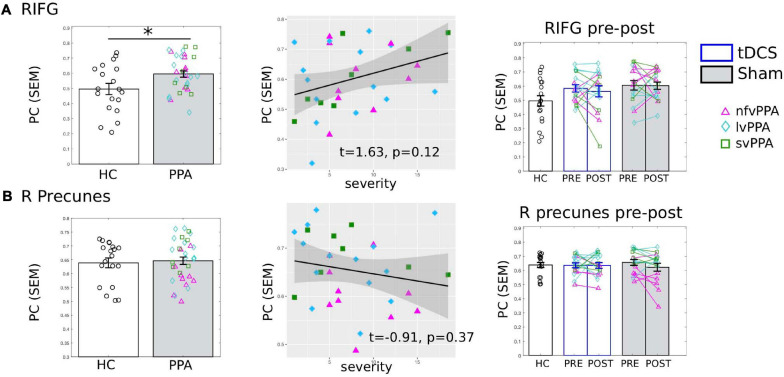
Global connectivity (*participation coefficient*, PC) of other control regions of interest (ROIs). Left to right: Comparison between HC and PPA at pre-treatment (both tDCS and sham combined); Relationship between pre-treatment PC and clinical dementia severity (FTDL-CDR); Pre- and post-treatment PC values for the three groups. The scatter plot depicts the effect from the multiple regression model and the data-points indicate the partial residuals (visualized with *jtools* in R). The three variants are visualized with different colors and markers. ^∗^*p* < 0.05. SEM, standard error of the mean. **(A)** The right inferior frontal gyrus triangularis (RIFG-tri). Similar to the LIFG-tri (shown in Figure 3), at pre-treatment, the PPA group had significantly higher PC values than the HCs (*p* = 0.01) and there was a (trending) association between PC and dementia severity. In contrast with the LIFG-tri (Figure 3), there were no pre-post treatment changes for either group (tDCS *p* = 0.56, Sham *p* = 0.93, interaction *p* = 0.71). **(B)** The right precuneus. There was no difference in PC values between HC and PPA groups (*p* = 0.73) at pre-treatment, and PC values did not correlate with dementia severity. There were no pre-post treatment changes for either group (tDCS *p* = 0.97, Sham *p* = 0.13, interaction *p* = 0.33).

To evaluate whether the connectivity effects documented in the previous analyses for the tDCS target LIFG-tri were regional specific, we examined the PC values of “control” ROIs. First, we examined the right-hemisphere homolog, i.e., the right RIFG-tri. At the pre-treatment time-point, as we reported for the LIFG-tri, the RIFG-tri also showed higher than normal PC in PPA (*t* = 2.62, *p* = 0.0119), and a positive correlation between PC and dementia severity that trended toward significance (FTDL-CDR, *t* = 1.63, *p* = 0.12, Figure 6A). No other variables showed significant effects (Supplementary Material 1). Regarding pre-post changes for the RIFG-tri, no difference between the two treatment-groups was found (*t* = −0.79, *p* = 0.44), and neither group showed pre-post changes [tDCS *t*(15) = −0.59, *p* = 0.56; Sham *t*(15) = 0.09, *p* = 0.93, Figure 6A, see also Supplementary Material 1]. The results indicate that although the left and right IFG shared the same abnormal global hyper-connectivity, the right IFG’s connectivity did not change following stimulation to the left IFG.

#### The Right Precuneus

We also evaluated the right precuneus which we hypothesized to be a more neutral ROI than the right IFG because: (1) it is typically considered to be the center of the default-mode network (DMN) and thus belongs to a different module than the IFG and (2) the region is relatively spared in PPA. Consistent with this hypothesis, HC and PPA groups did not differ at pre-treatment (tDCS and Sham combined, *t* = 0.88, *p* = 0.39) and the PC values of the right precuneus did not correlate with dementia severity (FTDL-CDR, *t* = −0.91, *p* = 0.37) or other variables (Figure 6B, see also Supplementary Material 1). Moreover, in terms of pre-post treatment changes, no differences between the two treatment-groups were found (*t* = 1.01, *p* = 0.32), and neither group changed from pre- to post-treatment [tDCS *t*(15) = 0.04, *p* = 0.97, Sham *t*(15) = 1.59, *p* = 0.13, Figure 6B].

#### The Other LIFG Subdivisions

Although we aimed to target the triangularis subdivision, given that tDCS current cannot be precisely directed and there is current spread (see Figure 1B for the current flow modeling), we also examined if there were treatment-induced changes in the other two adjacent LIFG subdivisions: the more ventral LIFG-*orbitalis* and the more dorsal/posterior LIFG-*opercularis*.

**FIGURE 7:**
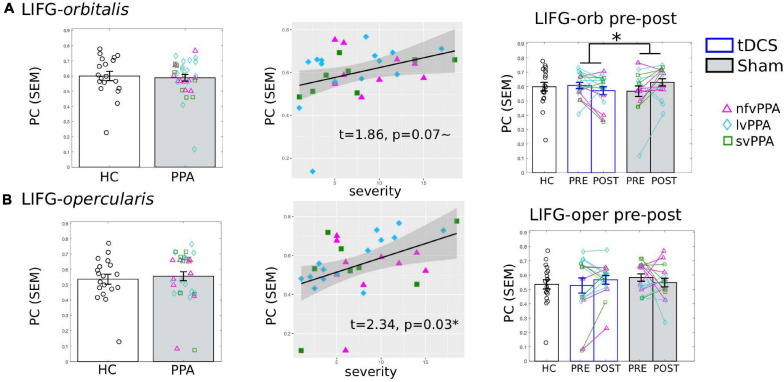
Global connectivity (*participation coefficient*, PC) of other left inferior frontal gyrus subdivisions. Left to right: Comparison between HC and PPA at pre-treatment (both tDCS and sham combined); Relationship between pre-treatment PC and clinical dementia severity (FTDL-CDR); Pre- and post-treatment PC values for the three groups. The scatter plot depicts the effect from the multiple regression model and the data-points indicate the partial residuals (visualized with *jtools* in R). The three variants are visualized by different colors and markers. *p < 0.05. SEM, standard error of the mean. **(A)** The LIFG-orbitalis. There was no difference in PC values between HC and PPA groups (*p* = 1.0) at pre-treatment. However, similar to the LIFG-triangularis, there was a marginal correlation between PC and dementia severity. And also similar to the LIFG-tri, there is a significant interaction between treatment-group and time-point (*p* = 0.05), although the changes for either group do not reach statistical significance (tDCS: *p* = 0.19, Sham *p* = 0.06). **(B)** The LIFG-opercularis. There was no difference in PC values between HC and PPA groups (*p* = 0.99) at pre-treatment, though there was an association between PC and dementia severity, similar to the other LIFG subdivisions. There were no pre- and post-treatment changes for either group (tDCS *p* = 0.24, Sham *p* = 0.4, interaction *p* = 0.19).

The results showed that the LIFG-*orbitalis* exhibited generally similar, but smaller and non-significant effects as the targeted LIFG-tri. Specifically, before treatment, although HC and PPA groups did not differ (pre-treatment, tDCS and Sham combined, *t* = 0.004, *p* = 1.0), the PPA’s PC values were marginally correlated with dementia severity (FTDL-CDR, *t* = 1.86, *p* = 0.07, Figure 7A) and no other variables showed a significant correlation (Supplementary Material 1). Regarding pre-post treatment changes, the LIFG-*orbitalis* also showed a similar effect as the targeted LIFG-tri such that the two treatment-groups differed from each other (*t* = −2.10, *p* = 0.049), driven by a decrease in connectivity of the tDCS group and an increase in the Sham group (Figure 7A), though the effects did not reach significance for either group [tDCS *t*(15) = −1.36, *p* = 0.1946; Sham *t*(15) = 2.02, *p* = 0.0614].

For the LIFG-*opercularis*, similar to the LIFG-*orbitalis*, PPA also did not differ from HC before treatment (*t* = 0.50, *p* = 0.62), although its PC values were significantly positively correlated with dementia severity (FTDL-CDR, *t* = 2.34, *p* = 0.03, Figure 7B). However, the LIFG-*opercularis* did not show any significant treatment-related changes [difference between tDCS and Sham: *t* = 1.34, *p* = 0.19; pre- to post-changes: tDCS *t*(15) = 1.22, *p* = 0.24, Sham *t*(15) = −0.86, *p* = 0.40, Figure 7B].

In sum, we did not observe consistent effects in the other LIFG subdivisions despite their vicinity to the stimulation site, indicating considerable regional specificity of the tDCS effects. Given the unspecific tDCS current spread (which should have extended to these adjacent regions), the observed regional specificity may have been driven by the pairing between the stimulation site and the targeted cognitive function. We will discuss the interpretation of these findings further in the General Discussion.

### Motion Artifacts

The amount of in-scanner motion of each individual was measured as the root-mean-squared (rms) of the six motion parameters and was included in all the regression analyses (see Supplementary Materials 2). There was no significant difference in the amount of motion between the HC and the PPA groups at either time-point [Pre: *t*(49) = 0.06, *p* = 0.95; Post: *t*(49) = 0.64, *p* = 0.52], nor between the tDCS and the Sham groups at either time-point [Pre: *t*(30) = 1.22, *p* = 0.23; Post: *t*(30) = 0.48, *p* = 0.64]. Motion also did not differ between the two time-points for either the tDCS [*t*(15) = 0.71, *p* = 0.49] nor the Sham group [*t*(15) = 0.48, *p* = 0.64], and the differences between pre to post treatment motion parameters did not differ across the two groups [*t*(30) = 0.13, *p* = 0.90]. In terms of outlier volume rejection (i.e., scrubbing), only two HCs participants and six PPA participants (three tDCS and three Sham) had fMRI volumes marked as outliers which were rejected before calculating the connectivity values (see Supplementary Figure 3).

## Discussion

In this study, we examined tDCS-induced FC changes in a tDCS-augmented language intervention study of PPA, a neurodegenerative syndrome with language as the primary clinical manifestation. Anodal tDCS was applied to the LIFG area as an adjuvant to written naming treatment (Figure 1). tDCS-induced FC changes of the LIFG-tri, a region that plays a key role in lexical processing (Bookheimer, 2002; Vigneau et al., 2006), were evaluated via comparisons with sham stimulation as well as with HCs. Specifically, we focused on the extent of global connectivity, as indexed by the network measure PC (Guimerà and Amaral, 2005, Eq. 1) which quantifies the extent to which a region or node (e.g., the LIFG-tri) is connected across multiple modules. The key findings were as follows: (1) Before treatment, elevated global connectivity values of the LIFG-tri were associated with greater clinical dementia severity in PPA (Figure 3A); (2) After treatment, the tDCS group but not the Sham group, exhibited a significant decrease in the global connectivity of the LIFG-tri (Figure 3B), indicating a tDCS-induced normalization. More specifically, the decrease was driven by a reduction in the LIFG-tri’s *between-module* connections (Figures 4, 5). In other words, as a result of tDCS, the LIFG-tri became less connected with regions outside its own module *(perisylvian)*. In particular, the largest connectivity reductions occurred between the LIFG-tri and the *frontoparietal* (FP) module as well as the *temporal* module (Figure 5B); (3) The tDCS-induced changes in global connectivity were specific to the stimulation target LIFG-tri, as there were no consistently reliable effects for other “control” ROIs, including the RIFG and the precuneus (Figure 6), as well as other LIFG subdivisions (Figure 7). In sum, the results indicate that tDCS applied to the LIFG may increase system segregation in a manner that is manifested by a reduction in the LIFG-tri’s connectivity across multiple modules, a reversal of the abnormal connectivity pattern observed before treatment. Such changes may reflect more efficient, automatic cognitive processing of lexical semantic retrieval promoted by tDCS which may have the effect of requiring less involvement of (greater segregation from) other cognitive processes, such as attention and executive control, that may be carried out by the other modules.

### Anodal tDCS Enhances System Segregation and Increases Processing Efficiency of the LIFG Triangularis

The LIFG is one of the most consistently activated regions during language processing and considered to be a “language hub” (Hagoort, 2005; Vigneau et al., 2006). However, in terms of FC, the LIFG has not been typically found to be a “well-connected” region in the functional connectome literature (e.g., He et al., 2009; Bullmore and Sporns, 2012; Power et al., 2013; Ellenblum et al., 2019). This disparity between the LIFG’s task-evoked activation and FC profile may reflect a trade-off between specialization and flexibility, such that while the LIFG may be specialized in language processing, this specialization is achieved at the cost of reduced interaction with other systems.

Consistent with the hypothesis that the perisylvian language module’s high degree of segregation favors efficient language performance, in the current study we found that the LIFG-tri tended to be less segregated and more broadly connected across the whole brain in PPA than in HCs. This higher than normal global connectivity (*PC*) observed in the PPA individuals was associated with greater dementia severity (Figure 3A), indicating that the increased global connectivity of the LIFG-tri was a pathological change with detrimental behavioral consequences. After tDCS, we observed a reversal of this hyper-connectivity for the tDCS but not for the Sham group (Figure 3B). Furthermore, when we examined the topographical distribution of the connections underpinning the global connectivity decrease of the tDCS group, we found that, before treatment, the *within-module* connectivity of the LIFG-tri (i.e., within the perisylvian module) did not differ from that of the HCs and remained unchanged after treatment while, in contrast, the *between-module* connectivity between the LIFG-tri and other modules was higher than normal at pre-treatment and decreased to normal levels following treatment (Figures 4, 5). These decreases were most evident for the LIFG-tri’s connectivity with the *frontoparietal* (FP) and the *temporal* modules (Figure 5B). Based on these findings, we hypothesize that tDCS targeting the LIFG-tri increases this region’s functional segregation from other modules, and that this reduction in the LIFG-tri’s pathological hyper-connectivity is associated with its increased language processing efficiency.

Anodal tDCS administered to the LIFG has also been shown to improve language performance and induce neural changes both in healthy participants (Holland et al., 2011; Meinzer et al., 2012, 2013) as well as in MCI (Meinzer et al., 2015). Interestingly, the authors in those studies also argued that the improved language performance was due to tDCS-induced increase in the processing efficiency of the LIFG. For instance, Meinzer et al. (2012) applied concurrent anodal tDCS (or sham stimulation) to the LIFG during a semantic fluency task in healthy young participants and found better performance during tDCS compared to sham stimulation. Most interestingly, they also found that the task-evoked activation in the ventral LIFG (similar to the LIFG-tri examined in the current study), was significantly lower in the tDCS compared to the sham condition [Holland et al. (2011) also showed a similar finding]. Furthermore, when they examined the RSFC, they found it to be higher for tDCS than sham within the left-lateralized perisylvian language regions and lower in other areas like the visual and the sensory-motor area. On the basis of these findings, the authors concluded that the behavioral benefits of anodal tDCS resulted from increased efficiency of the ventral LIFG, a functionally specialized region for lexical semantic knowledge retrieval. In follow-up experiments, the research group evaluated the tDCS effects in older individuals (Meinzer et al., 2013) and individuals with MCI (Meinzer et al., 2015), and reached a similar conclusion that tDCS promoted processing efficiency of the LIFG.

The hypothesis that tDCS increases processing efficiency is also generally consistent with proposed neurophysiological mechanisms of anodal tDCS. It has been proposed that anodal tDCS acts to modify synaptic plasticity and induce long-term potentiation in the stimulation area. These changes have been associated with learning in the brain (Stagg and Nitsche, 2011). As a result, tDCS may enhance learning (or re-learning) with the effect of increasing the efficiency of cognitive processing. This increased processing efficiency could result in the reduced fMRI activation reported in the abovementioned studies that examined on-line fMRI task-evoked activation during tDCS (Holland et al., 2011; Meinzer et al., 2012, 2013, 2015). In this study which examined off-line rs-fMRI activities (before and after a multi-session tDCS intervention), increased processing efficiency may have reduced the LIFG-tri’s interaction with other modules, as reflected by the observed reduction of between-module FC. Note that, however, this proposed mechanism does not preclude the possibility that tDCS may also change, directly or indirectly, the neural activities of other distant regions may also change simultaneously.

#### Distinct Relationships Between Neural Changes and Behavioral Improvement in tDCS and Sham

In this study, we found an intriguing interaction effect between the tDCS and the Sham groups with regard to the relationship between neural changes and behavioral changes (Figure 3C). Specifically, we found that in the tDCS group, the global connectivity value (PC) for the stimulation site (LIFG-tri) decreased significantly (Figure 3B), and that larger *decreases* were associated with greater treatment gains, whereas for the Sham group, although the PC change was not significant at the group level (Figure 3B), larger PC *increases* were associated with greater treatment gains (Recall that both tDCS and Sham received behavioral therapy and participants in both conditions showed behavioral improvement, but the behavioral effect was larger for the tDCS compared to the Sham condition.) One interpretation of the opposite directionality of the effects is that the neural mechanism(s) supporting the behavioral benefits in the tDCS-augmented therapy condition are distinct from those supporting the benefits observed with behavioral therapy alone (Sham condition). As discussed in the previous section, our hypothesis is that the decreased PC of the LIFG observed in tDCS indicates greater system segregation and processing efficiency of the LIFG. If that is correct, it may be that this effective neural response which is enabled by tDCS does not occur in the Sham condition. Instead, in that condition the brain may have to resort to alternative, suboptimal strategies such as recruiting other resources. Of course this account would need to be evaluated with more targeted investigation.

### The Regional Specificity of tDCS Effects

To understand the underlying mechanism/s of tDCS, one important question is the extent to which observed tDCS effects are specific to a stimulated area. To evaluate this question, we first examined the right hemisphere homolog of the LIFG-tri, i.e., RIFG-tri, assuming that if the stimulation effects were not restricted to the stimulation site, one prime candidate would be its homolog given the strong functional and structural connection between homolog regions. However, as shown in Figure 6A, although the RIFG-tri, like the LIFG-tri, also exhibited elevated global connectivity levels prior to treatment, it did not show significant pre- to post-treatment changes. Moreover, the effects seemed to be specifically associated with the LIFG triangularis subdivision and, to a lesser extent, with the other anterior LIFG subdivision – the IFG-orbitalis. We did not, however, observe consistently reliable effects for the more dorsal and posterior subdivision LIFG-opercularis, despite its proximity to the stimulation site (Figure 7B). Because the LIFG-orb showed similar effects as the LIFG-tri, we speculate that the two ventral anterior subdivisions are likely to be similar in terms of their connectivity profiles and cognitive functions, and are distinct from the more dorsal posterior LIFG-opercularis. A similar anterior/ventral – posterior/dorsal distinction has also been observed in the other studies that applied tDCS during a semantic task (Holland et al., 2011; Meinzer et al., 2012) discussed below.

There are several possible reasons for these apparent spatially selective effects, and we note that these are not necessarily mutually exclusive. One possibility is simply that tDCS current flow is sufficiently focal such that the major impact is reasonably spatially limited. In this study, it would have to have been limited to the LIFG-tri subdivision. However, this seems somewhat unlikely given the wide and unspecific current spread of tDCS (Brunoni et al., 2012, see Figure 1B for the current modeling results). Another possibility concerns the pairing of the behavioral treatment and stimulation site. It has been hypothesized that the effects of tDCS are maximal when the cognitive function of the stimulated area and the treatment task are well aligned, a mechanism that has been referred to as “functional specificity” (Bikson and Rahman, 2013). The proposal is that tDCS is most likely to affect the neuronal activities of the stimulated area if that area is actively engaged by the behavioral task during the stimulation. Therefore, given that the LIFG-tri plays a key role in selection during lexical-semantic retrieval (Vigneau et al., 2006), the target cognitive function of our behavioral treatment, the functional specificity hypothesis seems most consistent with the observed regional specificity effect (see further discussion in the next paragraph). Finally, another related hypothesis is that the FC changes are linked to neurotransmitter changes caused by anodal tDCS (e.g., reduction in the inhibitory neurotransmitter GABA), which have been shown to be regionally specific (Clark et al., 2011; Hunter et al., 2015; Harris et al., 2019). For example, a previous study by our group found that the tDCS group showed a significant decrease in GABA in the LIFG, something which was not seen either in the Sham group or in the control ROI right sensory-motor cortex (Harris et al., 2019).

Notably, similar regional specificity has been reported in other anodal tDCS studies that also stimulated the LIFG. Both of these prior studies also highlighted the hypothesis that the regional specificity effects resulted from an optimal pairing of task and stimulation site (Holland et al., 2011; Meinzer et al., 2012). In both studies, healthy participants received anodal tDCS (or sham stimulation) to the LIFG while performing a semantic task (picture naming or semantic fluency task). Subsequent tDCS-induced fMRI activation reduction was specifically found in the anterior/ventral part of the left IFG, similar to the LIFG-tri in the current study, and no changes were seen in the right IFG and regions in the vicinity of stimulation (e.g., the dorsal IFG, the precentral gyrus). Both studies attributed the regional specificity effect to the role that the anterior/ventral LIFG plays in semantic processing. Thus, this regional specificity effect is consistent with the “functional specificity” hypothesis that anodal tDCS increases the activity of the task-relevant neurons. Nevertheless, given the scarcity of research on this topic, further study is needed to further evaluate this hypothesis.

We should note that the regional specificity we observed does not necessarily contradict the notion often found in the neurostimulation literature that tDCS (and other neurostimulation methods) can induce network-wide changes distal to the stimulation site (e.g., see Pini et al., 2018 for a review). In fact, the decrease in the LIFG-tri’s global connectivity that we reported here did involve changes in connectivity between the LIFG-tri and various regions/modules across the brain (Figures 4, 5). However, given that, of all the ROIs examined, only the LIFG-tri (and to a lesser extent the LIFG-orbitalis) showed decreased overall global connectivity (PC), the results indicate that the widespread connectivity changes induced by tDCS are likely to stem from the ventral/anterior part of the LIFG, in particular the triangularis. In addition, secondary connectivity changes in other areas or networks are also possible. For instance, the decoupling between the LIFG-tri and the FP module that we observed might introduce additional changes within the FP module (that were not evaluated in this study).

### Limitations and Future Considerations

First, in the current study we focused on the stimulation site LIFG, hence our conclusion only apply to the LIFG (triangularis) and not to other regions or to the whole-brain network organization, which may respond differently to stimulation. Indeed, in a recent study (Tao et al., 2020) that examined the global, whole-brain network properties in PPA, we found that the average global integration across the brain was reduced relative to HCs (as measured by global network measures, such as *global efficiency* and *clustering coefficient*). Therefore, it is possible that such whole-brain characteristics, as well as other brain regions that play very different roles than the LIFG-tri, may show different tDCS-induced changes. Second, in this study, we focused on the PC measure which specifically evaluates a node’s system segregation level as the connectivity diversity across multiple modules (Eq. 1, also see Figure 4 for the modules used in this study). As a result, however, we can only draw conclusions regarding the aspect of system segregation that is quantified by PC. There are a number of other centrality measures that may capture different aspects of a node’s connectivity profile (e.g., *degrees*, *betweenness*, see review by Rubinov and Sporns, 2010) and which may provide additional understanding of the effects of tDCS on FC. A third limitation is the possibility that PPA variants may show different responses to LIFG tDCS. We did not examine this in the current study due to the relatively small sample sizes for each variant. One potential factor that could cause different effects across the PPA variants is that there may be differences in the tDCS current flow due to different distributions of atrophy across the variants. However, this issue was recently investigated by our group and we did not find consistent differences in current flow across the three variants (Unal et al., 2020). Nonetheless even if we assume similar current flow, the underlying disease characteristics across the variants might still play a role in determining neuro-stimulation outcomes. For instance, tDCS might have a larger impact when applied to an already compromised region compared to a healthy one. Thus, it is possible that participants of the non-fluent variant with substantial damage in the LIFG and regions functionally or anatomically connected to the LIFG would show the greatest tDCS benefit. Indeed, this possibility is consistent with results reported by Tsapkini et al. (2018) that the non-fluent variant showed the most robust treatment gains across both arms of the clinical trial (from which the current data set was drawn) and generalized well to untrained words, whereas the logopenic and semantic variants, whose atrophy is relatively distant from LIFG, showed less robust treatment gains. Furthermore, in another study we found that greater tDCS-induced treatment gains were associated with smaller gray matter volume of several left frontal-parietal areas that were functionally and/or structurally connected with LIFG (de Aguiar et al., 2020). Alternatively, it could be the case that tDCS exerts a greater effect when the underlying neural tissue is less (rather than more) damaged, which would also result in different responses to stimulation across the variants. In sum, the interaction between tDCS-induced neural changes and different subtypes of PPA is an important issue that should be examined in the future with larger sample sizes.

## Conclusion

In the current investigation we examined RSFC changes induced by anodal tDCS as an adjuvant to behavioral language therapy in PPA. We found that, compared to sham stimulation, tDCS applied to the LIFG reduced the LIFG (triangularis)’s connectivity across multiple modules. This resulted in enhanced segregation between the perisylvian language module and the other modules, reflecting a normalization of the LIFG-tri’s pre-treatment hyper-connectivity that was accompanied by an augmented treatment-induced improvement. Furthermore, we found that the neural and behavioral tDCS-induced changes were largely specific to the LIFG-tri, a region closely associated with the lexical semantic retrieval process targeted by the naming treatment, highlighting the importance of appropriately pairing the behavioral task used during treatment and the cognitive functions supported by the stimulation site. Given the scarcity of research regarding the neural responses to tDCS in disease treatment, in particular for PPA, our findings provide much needed empirical evidence and have applications for validating efficacy and designing future tDCS and other NIBS treatments.

## Data Availability Statement

Data requests should be addressed to the PI of this study KT (tsapkini@jhmi.edu) with proper justification. The analysis scripts are available on osf.io/yu29z.

## Ethics Statement

The studies involving human participants were reviewed and approved by Johns Hopkins Hospital and Johns Hopkins University Institutional Review Board. The patients/participants provided their written informed consent to participate in this study. Written informed consent was obtained from the individual(s) for the publication of any potentially identifiable images or data included in this article.

## Author Contributions

YT, BR, and KT contributed to conceptualization and design of the study. BF collected the data, organized the database, and performed the data analysis. YT performed the data analysis and wrote the first draft of the manuscript. ZW performed analysis on the behavioral data. BR and KT contributed to manuscript revision and read and approved the submitted version. All authors contributed to the article and approved the submitted version.

## Conflict of Interest

The authors declare that the research was conducted in the absence of any commercial or financial relationships that could be construed as a potential conflict of interest.

## Footnotes

1 According to a recent review by Cotelli et al. (2020) that examined tDCS-augmented language intervention studies in PPA, our current sample size is larger than any other studies thus far (*n* ranges from 5 to 18 except studies from our laboratory that examined a similar set of the current participants). Therefore although the sample size may be considered moderate, the current study represents an advancement in the literature on language intervention in PPA.

2 www.WongBakerFACES.org

3 https://mricloud.org

4 https://braingps.mricloud.org/t1prep

5 https://braingps.mricloud.org/fmri

6 https://www.nitrc.org/projects/artifact_detect
