# Supplementary material for: Selective Functional Network Changes Following tDCS-Augmented Language Treatment in Primary Progressive Aphasia
Source: Front Aging Neurosci. 2021 Jul 12;13:681043. doi: 10.3389/fnagi.2021.681043 (PMC8311858; doi:10.3389/fnagi.2021.681043)
Supplement: Supplementary file 1 [file Data_Sheet_1.docx]

**Supplementary materials**

**Selective Functional Network Changes following tDCS-augmented Language Treatment in Primary Progressive Aphasia**

Yuan Tao, Bronte Ficek, Zeyi Wang, Brenda Rapp, Kyrana Tsapkini

**Supplementary material 1. Behavioral results.**

The treatment effects of this double-blind, sham-controlled, crossover clinical trial have been reported in Tsapkini et al. (2018). Thirty-six participants were included in the previous reported, 11 of which were not included in the current analyses due to the lack of a full imaging dataset, and since the last study, 7 additional participants were added to the database, and in total 32 participants (16 tDCS and 16 Sham) were included in the current study.

We evaluated the behavioral effect of tDCS compared to Sham following the method in Tsapkini et al. (2018). Improvement on the trained words was evaluated at immediate post-treatment, 2-week, and 2-month follow-up (Fig. 1a). The *average treatment effect* (ATE) of tDCS over sham was calculated as *δ_(TDCS vs SHAM)_ = E[Y|T = tDCS] - E[Y|T =Sham],* where Y is the improvement score for each post-treatment time-point. The following covariates were included in the model: pre-treatment accuracy, PPA variant, number of treatment sessions, sex, age, years post onset of symptoms, and clinical dementia rating (FTDL-CDR) and the language sub-score.

The estimation of *average treatment effect* (ATE) was conducted using the Targeted Minimum Loss-Based Estimation (TMLE) method (van der Laan & Rose, 2011^[[1]](#footnote-1)^) and the TMLE R package (Gruber & van der Laan, 2012a^[[2]](#footnote-2)^), in order to achieve doubly robust estimate consistency and semiparametric locally efficiency as commonly practiced methods such as augmented inverse propensity score weighting (AIPW) estimators while allowing potentially more efficient (cross validation based) data-adaptive covariates adjustment (Rotnitzky et al., 2012; Gruber & van der Laan 2012b^[[3]](#footnote-3)^). As shown in Fig. S1, from pre- to immediate post-treatment, tDCS showed significant augmentative effect over Sham (estimate: 8.93, CI: 0.89 -16.98, p = 0.03), which was maintained at 2-month follow-up (estimate: 16.17, CI: 7.63 - 24.71, p = 0.0002). The ATE estimation, standard errors (SE), p-values, and 95% confidence intervals (CI) were reported in Table S1.


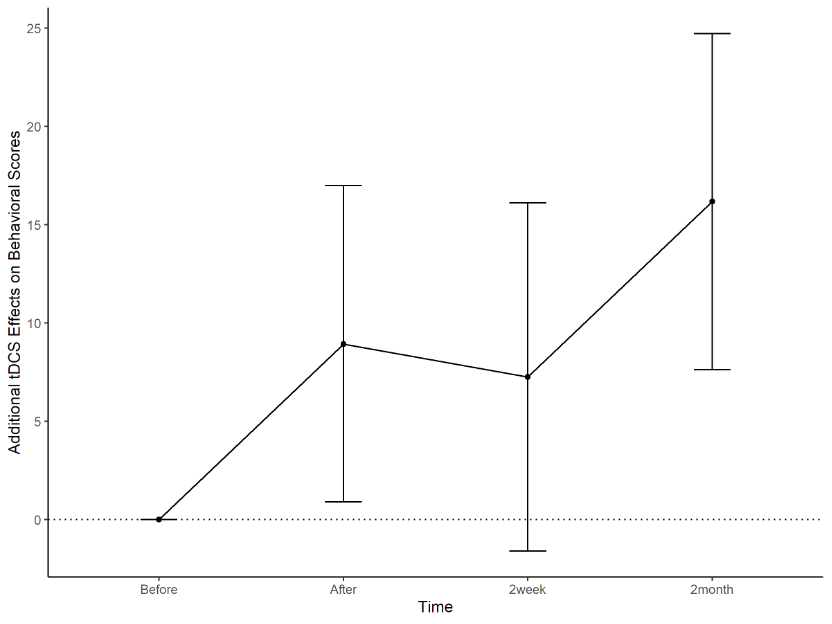


*

~

***

**Supplementary Figure 1**. The average treatment effect (ATE) estimates of tDCS over to Sham at immediate post-, 2-week, and 2-month follow-up time-points. Errorbars indicate 95% confidence intervals. Statistics are reported in Table S1. *p<0.05, ***p<0.0001, ~p<0.1.

**Supplementary Table 1. Causal effects of tDCS compared to Sham at the three post-treatment time-points.** Average treatment effect (ATE) estimates of tDCS over Sham, standard errors (SE), p-values, and 95% confidence intervals (CI) are reported.

|  | Estimate | SE | *P* | Lower | Upper |
| --- | --- | --- | --- | --- | --- |
| Immediate post | 8.93 | 4.11 | 0.030 | 0.89 | 16.98 |
| 2-week follow-up | 7.25 | 4.52 | 0.109 | -1.60 | 16.10 |
| 2-month follow-up | 16.17 | 4.36 | 2E-4 | 7.63 | 24.71 |

**Supplementary material 1. Results of multiple regression models** (see Section 2.7.2. *Statistical analyses on the network measure participation coefficient*). For each region-of-interest (ROI), three regression analyses were conducted: a) Comparison between healthy controls (HC) and PPA at pre-treatment (tDCS and sham combined); b) Relationship between PC and dementia severity at pre-treatment; c) Pre- to post-treatment changes. In all three models, the dependent variables were the ROI’s *participation coefficient* (PC) values. Detailed description of the models can be found in 2.7.2. Significant effects (p<0.05) are in bold. **: p<0.05, **: p<0.01, ***: p<0.001, ~: p<0.1.*

**Supplementary Table 2. Results of multiple regression models for each ROIs.**

1. **LIFG-triangularis (LIFG-tri)**

| a. HC vs. PPA at Pre-treatment | Est. coefficient | Std. error | t-value | p-value |
| --- | --- | --- | --- | --- |
| Participant group (PPA > HC) | 0.0354 | 0.0419 | 0.84 | 0.40 |
| Age | 0.0037 | 0.0028 | 1.34 | 0.19 |
| Years of education | 0.0049 | 0.0097 | 0.51 | 0.61 |
| Gender (Male > Female) | 0.0618 | 0.0425 | 1.45 | 0.15 |
| In-scanner motion | -0.0823 | 0.3435 | -0.24 | 0.81 |

| b. Pre-treatment | Est. coefficient | Std. error | t-value | p-value |
| --- | --- | --- | --- | --- |
| **Dementia severity (CDR_FTLD)** | **0.0169** | **0.0045** | **3.75** | **0.0009***** |
| Age | -0.0006 | 0.0035 | -0.16 | 0.87 |
| Years of education | -0.0095 | 0.0127 | -0.75 | 0.46 |
| **Gender (Male > Female)** | **0.0957** | **0.0418** | **2.29** | **0.03** |
| In-scanner motion | 0.1291 | 0.3735 | 0.35 | 0.73 |

| c. Change (Post – Pre) | Est. coefficient | Std. error | t-value | p-value |
| --- | --- | --- | --- | --- |
| **Treatment group (tDCS > Sham)** | **-0.0958** | **0.0403** | **-2.38** | **0.03*** |
| Dementia severity (CDR_FTLD) | -0.0084 | 0.0043 | -1.92 | 0.07~ |
| Age | -0.0023 | 0.0032 | -0.72 | 0.48 |
| Years of education | 0.0050 | 0.0108 | 0.46 | 0.65 |
| Gender (Male > Female) | 0.0022 | 0.0392 | 0.06 | 0.96 |
| In-scanner motion | 0.3054 | 0.2979 | 1.03 | 0.32 |

1. **RIFG-triangularis**

| a. HC vs. PPA at Pre-treatment | Est. coefficient | Std. error | t-value | p-value |
| --- | --- | --- | --- | --- |
| **Participant group (PPA > HC)** | **0.1056** | **0.0403** | **2.62** | **0.01*** |
| Age | 0.0005 | 0.0027 | 1.76 | 0.09~ |
| Years of education | -0.0074 | 0.0093 | -0.79 | 0.43 |
| Gender (Male > Female) | -0.0390 | 0.0409 | -0.95 | 0.35 |
| In-scanner motion | -0.4406 | 0.3303 | -1.33 | 0.19 |

| b. Pre-treatment | Est. coefficient | Std. error | t-value | p-value |
| --- | --- | --- | --- | --- |
| Dementia severity (CDR_FTLD) | 0.0080 | 0.0049 | 1.61 | 0.12 |
| Age | 0.0038 | 0.0038 | 1.63 | 0.32 |
| Years of education | -0.0058 | 0.0138 | 1.02 | 0.68 |
| Gender (Male > Female) | -0.0118 | 0.0453 | -0.42 | 0.80 |
| In-scanner motion | -0.2529 | 0.4048 | -0.26 | 0.54 |

| c. Change (Post – Pre) | Est. coefficient | Std. error | t-value | p-value |
| --- | --- | --- | --- | --- |
| Treatment group (tDCS > Sham) | -0.0429 | 0.0545 | -0.79 | 0.44 |
| Dementia severity (CDR_FTLD) | 0.0018 | 0.0059 | 0.31 | 0.76 |
| Age | -0.0052 | 0.0044 | -1.19 | 0.25 |
| Years of education | 0.0022 | 0.0147 | 0.15 | 0.88 |
| Gender (Male > Female) | 0.0399 | 0.0532 | 0.75 | 0.46 |
| In-scanner motion | 0.1845 | 0.4038 | 0.46 | 0.65 |

1. **Right precuneus**

| a. HC vs. PPA at Pre-treatment | Est. coefficient | Std. error | t-value | p-value |
| --- | --- | --- | --- | --- |
| Participant group (PPA > HC) | 0.0199 | 0.0227 | 0.88 | 0.39 |
| Age | -0.0028 | 0.0016 | -1.88 | 0.07~ |
| Years of education | -0.0050 | 0.0053 | -0.94 | 0.35 |
| Gender (Male > Female) | -0.0187 | 0.0231 | -0.81 | 0.42 |
| In-scanner motion | 0.1255 | 0.1863 | 0.67 | 0.50 |

| b. Pre-treatment | Est. coefficient | Std. error | t-value | p-value |
| --- | --- | --- | --- | --- |
| Dementia severity (CDR-FTLD) | -0.0030 | 0.0033 | -0.91 | 0.37 |
| Age | -0.0018 | 0.0025 | -0.73 | 0.48 |
| Years of education | -0.0016 | 0.0093 | -0.18 | 0.86 |
| Gender (Male > Female) | -0.0152 | 0.0305 | -0.50 | 0.62 |
| In-scanner motion | 0.0499 | 0.2730 | 0.18 | 0.86 |

| c. Change (Post – Pre) | Est. coefficient | Std. error | t-value | p-value |
| --- | --- | --- | --- | --- |
| Treatment group (tDCS > Sham) | 0.0388 | 0.0384 | 1.01 | 0.32 |
| Dementia severity (CDR_FTLD) | -0.0017 | 0.0041 | -0.42 | 0.68 |
| Age | 0.0010 | 0.0031 | 0.33 | 0.74 |
| Years of education | -0.0064 | 0.0103 | -0.61 | 0.54 |
| Gender (Male > Female) | -0.0225 | 0.0375 | -0.69 | 0.56 |
| In-scanner motion | -0.2014 | 0.2845 | -0.71 | 0.49 |

1. **LIFG-orbitalis**

| a. HC vs. PPA at Pre-treatment | Est. coefficient | Std. error | t-value | p-value |
| --- | --- | --- | --- | --- |
| Participant group (PPA > HC) | 0.0002 | 0.0391 | 0.004 | 1.00 |
| Age | -0.001 | 0.0026 | -0.39 | 0.70 |
| Years of education | 0.001 | 0.0090 | 0.12 | 0.90 |
| Gender (Male > Female) | -0.0365 | 0.0397 | -0.92 | 0.36 |
| In-scanner motion | -0.1912 | 0.3204 | -0.60 | 0.56 |

| b. Pre-treatment | Est. coefficient | Std. error | t-value | p-value |
| --- | --- | --- | --- | --- |
| Dementia severity (CDR-FTLD) | 0.0092 | 0.0050 | 1.86 | 0.07~ |
| Age | -0.0006 | 0.0038 | -0.17 | 0.87 |
| Years of education | -0.0085 | 0.0140 | -0.61 | 0.55 |
| Gender (Male > Female) | -0.0263 | 0.0459 | -0.57 | 0.57 |
| In-scanner motion | -0.3318 | 0.4109 | -0.81 | 0.43 |

| c. Change (Post – Pre) | Est. coefficient | Std. error | t-value | p-value |
| --- | --- | --- | --- | --- |
| **Treatment group (tDCS > Sham)** | **-0.0967** | **0.0467** | **-2.07** | **0.05*** |
| Dementia severity (CDR_FTLD) | -0.0007 | 0.0050 | -0.14 | 0.89 |
| Age | 0.0006 | 0.0037 | 0.15 | 0.88 |
| Years of education | -0.0008 | 0.0126 | -0.07 | 0.95 |
| Gender (Male > Female) | 0.0663 | 0.0456 | 1.46 | 0.16 |
| In-scanner motion | 0.0601 | 0.3459 | 0.18 | 0.86 |

1. **LIFG-opercularis**

| a. HC vs. PPA at Pre-treatment | Est. coefficient | Std. error | t-value | p-value |
| --- | --- | --- | --- | --- |
| Participant group (PPA > HC) | 0.0241 | 0.0484 | 0.50 | 0.62 |
| Age | 0.0024 | 0.0032 | 0.75 | 0.46 |
| Years of education | 0.0040 | 0.0112 | 0.36 | 0.72 |
| Gender (Male > Female) | -0.0383 | 0.0491 | -0.78 | 0.44 |
| In-scanner motion | 0.4756 | 0.3966 | 1.20 | 0.24 |

| b. Pre-treatment | Est. coefficient | Std. error | t-value | p-value |
| --- | --- | --- | --- | --- |
| **Dementia severity (CDR-FTLD)** | **0.0147** | **0.0063** | **2.34** | **0.03*** |
| Age | -0.0031 | 0.0049 | -0.64 | 0.53 |
| Years of education | 0.0117 | 0.0177 | 0.66 | 0.51 |
| Gender (Male > Female) | 0.0040 | 0.0582 | 0.07 | 0.95 |
| **In-scanner motion** | **1.0831** | **0.5210** | **2.08** | **0.05*** |

| c. Change (Post – Pre) | Est. coefficient | Std. error | t-value | p-value |
| --- | --- | --- | --- | --- |
| Treatment group (tDCS > Sham) | 0.0680 | 0.0506 | 1.34 | 0.19 |
| **Dementia severity (CDR_FTLD)** | **-0.0167** | **0.0055** | **-3.05** | **0.0054**** |
| Age | 0.0008 | 0.0040 | 0.19 | 0.85 |
| Years of education | 0.0012 | 0.0136 | 0.09 | 0.93 |
| Gender (Male > Female) | 0.0347 | 0.0493 | 0.70 | 0.49 |
| In-scanner motion | 0.4425 | 0.3746 | 1.18 | 0.25 |

**Supplementary Table 3. Results of multiple regression model examining the relationship between connectivity changes of the LIFG-triangularis and behavioral changes** (see Section 3.3.3 and Fig. 3c). Dependent variable is the treatment-related behavioral changes. The variable “PC changes” is the PC difference between the two time-points (Post subtracts Pre). Significant effects (p<0.05). **: p<0.05, ~: p<0.1.*

| Pre to post behavioral change | Est. coefficient | Std. error | t-value | p-value |
| --- | --- | --- | --- | --- |
| PC changes | 1.5564 | 0.7593 | 2.05 | 0.05~ |
| Treatment group (tDCS > Sham) | 0.1747 | 0.1254 | 1.39 | 0.18 |
| **Dementia severity (CDR_FTLD)** | **-0.036** | **0.0131** | **-2.75** | **0.01*** |
| Age | 0.0041 | 0.0091 | 0.45 | 0.66 |
| Years of education | -0.0116 | 0.0278 | -0.42 | 0.68 |
| Gender (Male > Female) | -0.0224 | 0.1081 | -0.21 | 0.84 |
| **PC changes : Treatment group** | **-2.4948** | **1.0051** | **-2.48** | **0.02*** |

**Supplementary Material 3. Comparisons the LIFG-tri’s connectivity by module** (see 2.7.3 *Examining within-module and between-module connectivity of the LIFG*). For each module, three comparisons were conducted: Pre to post-treatment changes of the tDCS group (paired t-test) and comparisons between healthy controls (HC) and the tDCS group at pre- and post-treatment (independent t-tests). T-values are shown in the table and effects of the *between-module* connectivity were corrected with FDR correction for the 6 modules (Benjamini and Hochberg, 1995), significant effects are in bold. See Section 2.7.3, 3.3.4, and Fig. 5b and Fig. S1. **:p<0.05, **:p<0.01, ~:p<0.1.*

**Supplementary Table 4. Statistics (t-values and significance) of the *within- and between-module* connectivity effect.**

|  | tDCS | | | Sham | | |
| --- | --- | --- | --- | --- | --- | --- |
|  | Pre-post change  (positive: Post > Pre) | HC vs. Pre  (positive: PPA > HC) | HC vs. Post  (positive: PPA > HC) | Pre-post change  (positive: Post > Pre) | HC vs. Pre  (positive: PPA > HC) | HC vs. Post  (positive: PPA > HC) |
| *Perisylvian*  *(within-module FC)* | 0.36 | -0.32 | -0.08 | 0.82 | 0.41 | 1.24 |
| *Temporal* | -**3.22*** | 2.01 | -0.21 | 1.93 | -1.02 | 0.61 |
| *FP* | -2.22 | **3.14*** | 1.44 | 1.11 | 1.72 | **3.01*** |
| *dFP* | -1.36 | -0.38 | -1.31 | -1.18 | 0.31 | -0.36 |
| *vmPFC* | -0.63 | 1.18 | 0.41 | -0.46 | 0.89 | 0.71 |
| *Occipital-temporal* | -1.34 | 0.75 | -0.74 | -0.31 | -0.07 | -0.42 |
| *Subcortical* | 1.41 | 1.29 | 1.99 | 0.90 | -0.25 | 0.65 |

**Left-hemisphere (LH):**

|  | tDCS | |  | Sham | | |
| --- | --- | --- | --- | --- | --- | --- |
|  | Pre-post change  (positive: Post > Pre) | HC vs. Pre  (positive: PPA > HC) | HC vs. Post  (positive: PPA > HC) | Pre-post change  (positive: Post > Pre) | HC vs. Pre  (positive: PPA > HC) | HC vs. Post  (positive: PPA > HC) |
| *Perisylvian*  *(within-module FC)* | 0.52 | -0.70 | -0.37 | 0.35 | -0.02 | 0.32 |
| *Temporal* | -1.47 | 1.08 | -0.02 | 2.35 | 1.49 | 0.42 |
| *FP* | -1.92 | **3.25*** | 1.51 | 0.50 | 2.47 | 3.05 |
| *dFP* | -0.97 | -0.67 | -1.30 | -2.83 | 0.61 | -0.86 |
| *vmPFC* | -0.70 | 1.27 | 0.39 | -0.26 | 0.88 | 0.83 |
| *Occipital-temporal* | -1.47 | 0.91 | -0.65 | -0.27 | -0.53 | -0.78 |
| *Subcortical* | *2.76~* | -0.35 | 1.52 | 1.39 | -1.39 | 0.02 |

**Right-hemisphere (RH):**

|  | tDCS | | | SHAM | | |
| --- | --- | --- | --- | --- | --- | --- |
|  | Pre-post change  (positive: Post > Pre) | HC vs. Pre  (positive: PPA > HC) | HC vs. Post  (positive: PPA > HC) | Pre-post change  (positive: Post > Pre) | HC vs. Pre  (positive: PPA > HC) | HC vs. Post  (positive: PPA > HC) |
| *Perisylvian*  *(within-module FC)* | 0.22 | -0.04 | 0.13 | 0.99 | 0.61 | 1.60 |
| *Temporal* | **-4.31**** | **2.83*** | -0.49 | 1.02 | -0.27 | 0.71 |
| *FP* | -2.27 | **2.43*** | 1.10 | 1.84 | 0.86 | 2.34 |
| *dFP* | -1.45 | -0.06 | -1.15 | 0.34 | -0.06 | 0.17 |
| *vmPFC* | -0.44 | 0.83 | 0.38 | -0.61 | 0.81 | 0.23 |
| *Occipital-temporal* | -1.16 | 0.50 | -0.77 | -0.33 | 0.41 | -0.004 |
| *Subcortical* | -0.14 | **3.37*** | 2.29 | 0.23 | 1.29 | 1.37 |

****Supplementary Figure 2.** LIFG-triangularis connections by module and by hemisphere (corresponding to Fig. 5b). The number of connections of the LIFG-tri for the healthy control group (HC, black) and the PPA group before and after tDCS treatment (gray and blue respectively) are shown. The values of the Perisylvian module on the leftmost position indicates within-module connections as the LIFG-tri is in the perisylvian module, and the others are between-module connections. The statistics are reported in Supplementary Material 2. *p<0.05, **:p<0.01. ~: p<0.1

**

**Supplementary Figure 3.** Motion artifacts. Left: The root-mean-squared (rms) measured from the six motion parameters of each individual and time-point. There is difference between groups and time-points (see Section 3.5 in the main text). Right: Numbers of rejected outlier volumes of each PPA participants at pre and post time-point (tDCS: blue star; Sham: black circle).

a) b)

HC

PPA

- 0.05

- 0.1

- 0.2

- 0.25

- 0.3

- 0.4

c) d)

**Supplementary Figure 4.** Effects of the PC values of LIFG-triangularis across the proportional threshold values (5% to 40%). The effects correspond to the effects shown in Fig. 3 in the main text. a) Pre-treatment PC values of the PPA participants (tDCS and Sham group combined, gray) compared to the healthy control group (HC, white). PPA shows higher value than HC consistently across the threshold values. b) Relationship between the pre-treatment PC and the clinical dementia rating score (CDR), values at each proportional threshold are indicated by different colors. c) and d) Pre- and post-treatment PC values of the tDCS (blue) and the Sham group (black) respectively. For tDCS, pre-treatment values (circle) are higher value than post-treatment values (star) consistently across the threshold values, and there is no clear difference for Sham.


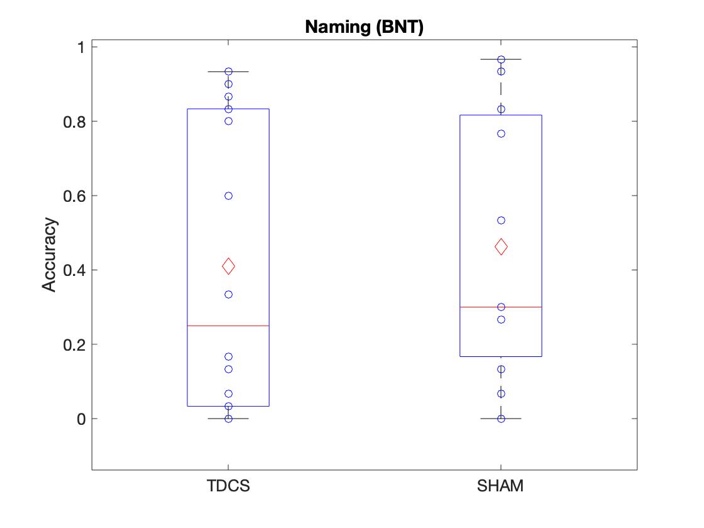

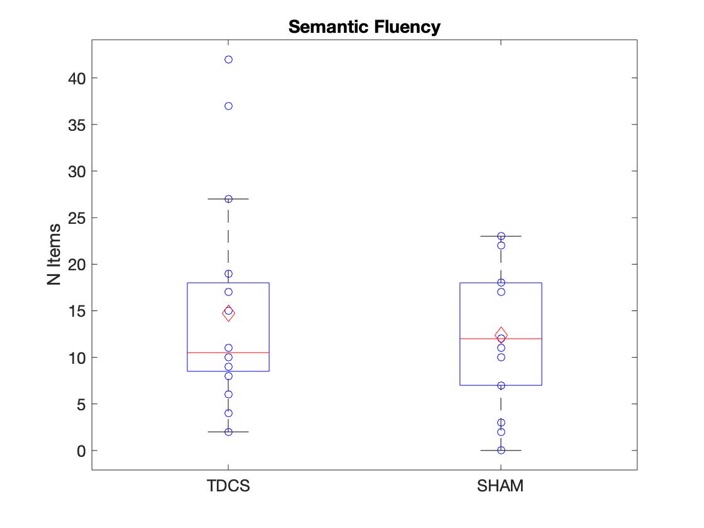

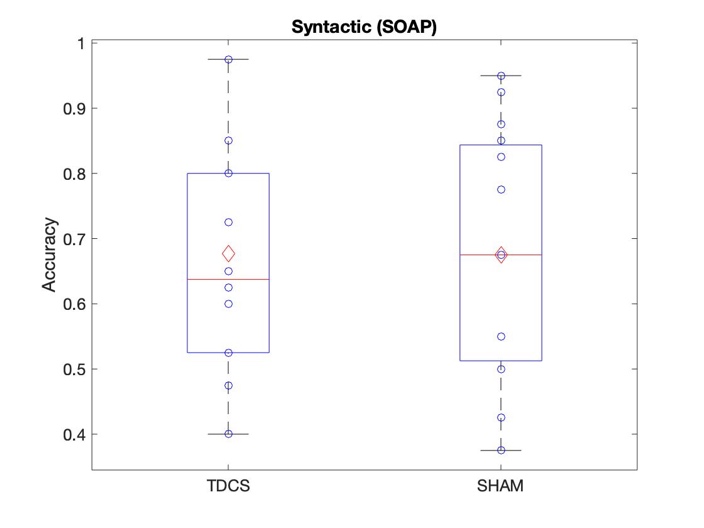


**Supplementary Figure 5.** Comparisons between the tDCS and the Sham group in other language tasks that are closely related to the LIFG-tri: Naming (Boston Naming Test), semantic fluency, and syntactic processing (SOAP). The horizontal lines indicate the median and the diamonds indicate the mean. No difference is found between the two treatment groups (BNT: t=-0.39, p=0.7; semantic fluency: t=0.59, p=0.49; SOAP: t=0.03, p=0.98).

**Supplementary Material 4. Examining age effects.**

As the tDCS and the Sham group showed a significant difference in age (Table 1), to make sure the observed effects were not simply due to this age difference, we repeated the analyses for the main effects with subset of the participants. Specifically, we excluded 3 participants from the tDCS group who were younger than 55 years old, and 2 from the Sham group who were older than 75, resulting in matched age between the two groups (t(15)=0.61, p=0.55, Fig. S6).

We repeated the analyses for the main effects found with the LIFG-tri as reported in Section 3.3 and Fig.3 for the remaining 27 PPA participants (13 tDCS and 14 Sham). As with the whole sample, at pre-treatment, higher PC values were associated with higher dementia severity (FTDL-CDR, t=3.25, p=0.0039, Fig. S7a), indicating that the higher global connectivity of the LIFG-tri might be a result of the disease. In addition males had higher PC values than females (t=2.28, p=0.0311), and no other variables showed significant effects (Table S5b).

Regarding the treatment-related changes, as with the whole sample, we found a significant difference in the magnitude of the changes between the two treatment groups (t=-2.13, p=0.0456, Fig. S7b). None of the other variables (i.e., age, year of education, gender, dementia severity, in-scanner motion) showed significant effects (see Table S5c). And similar to the whole sample, the difference was specifically driven by a marginally significant decrease in the tDCS group (t(12)=-2.48, p=0.0823), in contrast, the Sham group’s PC values did not change from pre- to post-treatment (t(13)=-1.05, p=0.31).


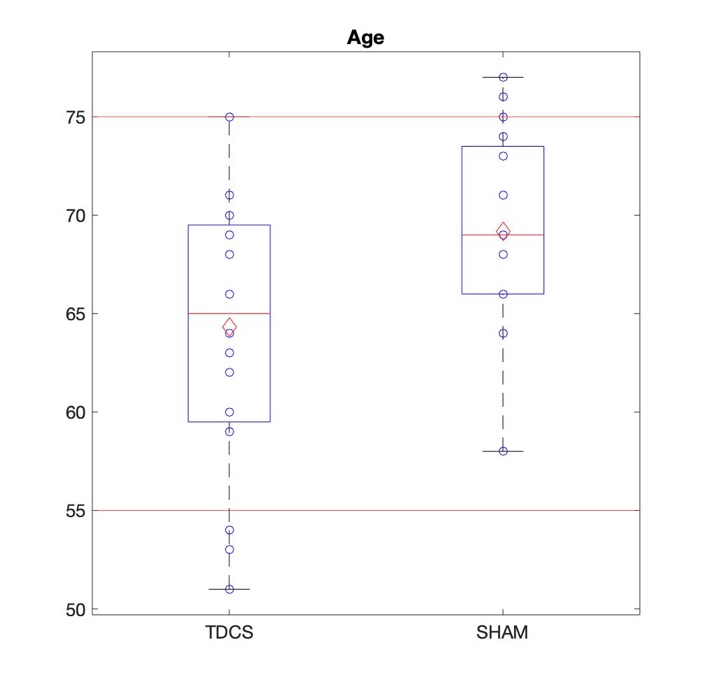
 Regarding the relationship between PC changes and the treatment-related behavioral changes, as with the original sample, we found a significant interaction (t=3.04, p=0.0078, Fig. S7c) between the two groups such that for the tDCS group, greater PC decrease was associated with greater treatment gain (r=-0.43, p=0.08), whereas the Sham group showed the opposite pattern (r=0.75, p=0.0006).

**Supplementary Figure 6. Age of the tDCS group and the Sham group.** For the analyses in Supplementary Material 4, with participants that are between 55 and 75 years old (indicated by the read cut-off lines).

**Supplementary Figure 7.** Repeating analyses with subsets of the original sample that the tDCS (n=13) and Sham (n=14) matched in age. Global connectivity (participation coefficient, PC) of the tDCS stimulation target left inferior frontal gyrus, triangularis (LIFG-tri) is examined. The figure corresponds to Fig. 3 in the main text. **a)** Pre-treatment PC values of the PPA participants (both tDCS and Sham group combined) compared to the healthy controls (HC). Left: PPA shows numerically higher PC than HC though the difference is not statistically significant. Right: Higher PC is associated with behaviorally measured dementia severity (FTDL-CDR. Higher values indicate greater severity). The plot depicts the partial residuals of the regression model (visualized with jtools in R). The three variants are visualized by different colors and shapes. **b)** Pre- to post- treatment changes of the tDCS group and the Sham group, values of the HC are the same as in a). The tDCS and the Sham group differ significantly in terms of pre- to post-treatment PC changes, driven by a significant PC decrease for the tDCS group with no change for the Sham group. The three variants are visualized by different colors and shapes as in a). **c)** Relationship between PC and behavioral changes. The plot depicts the partial residuals of the regression model (visualized with the package interactions in R). The tDCS and the Sham groups are indicated by blue and black respectively. The x-axis shows the PC changes (Post minus Pre), with positive values indicating increase from pre- to post-treatment and vice versa. The y-axis shows the treatment- related behavioral changes measured as proportional of maximal gain, such that the maximum improvement value is 100%. *p<0.05, ***p<0.001. SEM: standard error of the mean.

**Supplementary Table 5. Results of multiple regression model examining effects of the LIFG-triangularis for the 27 age-matched PPA participants (13 in tDCS and 14 in Sham)**. The tables are the same as in Table S1 (1) and Table S2. *: p<0.05, ~: p<0.1.

| a. HC vs. PPA at Pre-treatment | Est. coefficient | Std. error | t-value | p-value |
| --- | --- | --- | --- | --- |
| Participant group (PPA > HC) | 0.014 | 0.0441 | 0.32 | 0.75 |
| Age | 0.0067 | 0.0035 | 1.90 | 0.07~ |
| Years of education | 0.0088 | 0.0118 | 0.74 | 0.46 |
| Gender (Male > Female) | 0.0557 | 0.0469 | 1.19 | 0.24 |
| In-scanner motion | -0.0641 | 0.3786 | -0.17 | 0.87 |

| b. Pre-treatment | Est. coefficient | Std. error | t-value | p-value |
| --- | --- | --- | --- | --- |
| **Dementia severity (CDR_FTLD)** | **0.0164** | **0.0051** | **3.25** | **0.0039**** |
| Age | 0.0035 | 0.005 | 0.70 | 0.49 |
| Years of education | -0.0117 | 0.0184 | -0.63 | 0.53 |
| **Gender (Male > Female)** | **0.1151** | **0.0505** | **2.28** | **0.03*** |
| In-scanner motion | -0.012 | 0.4886 | -0.02 | 0.98 |

| c. Change (Post – Pre) | Est. coefficient | Std. error | t-value | p-value |
| --- | --- | --- | --- | --- |
| **Treatment group (tDCS > Sham)** | **-0.0967** | **0.0453** | **-2.13** | **0.0456*** |
| Dementia severity (CDR_FTLD) | -0.0067 | 0.0050 | -1.35 | 0.19 |
| Age | -0.0033 | 0.0048 | -0.68 | 0.51 |
| Years of education | 0.0019 | 0.0141 | 0.14 | 0.89 |
| Gender (Male > Female) | 0.0286 | 0.0475 | 0.60 | 0.55 |
| In-scanner motion | 0.3162 | 0.3729 | 0.85 | 0.41 |

| d. Pre to post behavioral change | Est. coefficient | Std. error | t-value | p-value |
| --- | --- | --- | --- | --- |
| **PC changes** | **1.4575** | **0.6825** | **2.14** | **0.0485*** |
| Treatment group (tDCS > Sham) | 0.1613 | 0.109 | 1.48 | 0.16 |
| **Dementia severity (CDR_FTLD)** | **-0.0311** | **0.0112** | **-2.76** | **0.01*** |
| Age | -0.0057 | 0.0103 | -0.55 | 0.60 |
| Years of education | -0.0077 | 0.0284 | -0.27 | 0.79 |
| Gender (Male > Female) | 0.0428 | 0.0978 | 0.44 | 0.67 |
| **PC changes : Treatment group** | **-2.7109** | **0.8925** | **-3.04** | **0.0078**** |

1. van der Laan, M., & Rose, S. (2011). *Targeted Learning: Prediction and Causal Inference for Observational and Experimental Data*. New York: Springer. [↑](#footnote-ref-1)
2. Gruber, S., & van der Laan, M. (2012). tmle: An R Package for Targeted Maximum Likelihood Estimation. *Journal of Statistical Software*, *51*(1), 1–35. https://doi.org/10.18637/jss.v051.i13 [↑](#footnote-ref-2)
3. Rotnitzky, A., Lei, Q., Sued, M., & Robins, J. M. (2012). Improved double-robust estimation in missing data and causal inference models. *Biometrika*, *99*(2), 439-456.

   Gruber, S., & van der Laan, M. J. (2012). Targeted minimum loss based estimator that outperforms a given estimator. *The International Journal of Biostatistics*, *8*(1). [↑](#footnote-ref-3)
